# An Updated 16-Year Pharmacovigilance Analysis of Neuropsychiatric Safety Profiles of Ciprofloxacin, Levofloxacin, and Moxifloxacin Using FAERS Data

**DOI:** 10.3390/ph19060820

**Published:** 2026-05-23

**Authors:** Aura Rusu, Ioana-Maria Stroia, Marius Călin Cherecheș

**Affiliations:** 1 Pharmaceutical and Therapeutic Chemistry Department, Faculty of Pharmacy, George Emil Palade University of Medicine, Pharmacy, Science, and Technology of Târgu Mureș, 540142 Târgu Mureș, Romania; aura.rusu@umfst.ro; 2 Marketing, Management and Sustainability Department, Faculty of Pharmacy, George Emil Palade University of Medicine, Pharmacy, Science and Technology of Târgu Mureș, 540142 Târgu Mureș, Romania; marius.chereches@umfst.ro

**Keywords:** fluoroquinolones, neuropsychiatric adverse reactions, pharmacovigilance, FAERS, antibiotic safety, side-effects

## Abstract

**Background/Objectives**: Fluoroquinolones (FQNs) are widely prescribed broad-spectrum antibiotics but are associated with central and peripheral nervous system adverse reactions (ARs). Regulatory agencies have issued multiple safety warnings regarding their neuropsychiatric effects; however, large-scale, comparative evaluations across individual FQNs remain limited. This study aimed to comprehensively characterise and compare neuropsychiatric profiles associated with Ciprofloxacin, Levofloxacin, and Moxifloxacin using pharmacovigilance data. **Methods**: A retrospective pharmacovigilance study was conducted using reports from the U.S. Food and Drug Administration Adverse Event Reporting System (FAERS) between 2010 and 2025. Neuropsychiatric ARs were identified using MedDRA terms, including neurological and psychiatric manifestations. Reporting trends, demographic characteristics, and event frequencies were descriptively analysed. Signal detection was performed using the Information Component (IC), Proportional Reporting Ratio (PRR), and Reporting Odds Ratio (ROR). **Results**: A total of 95,968 individual case safety reports involving neuropsychiatric ARs were included. Levofloxacin accounted for the highest number of reported events, followed by Ciprofloxacin and Moxifloxacin. Disproportionality analyses identified peripheral neuropathy as the strongest neurological signal for Levofloxacin and Moxifloxacin, while Ciprofloxacin showed stronger central nervous system associations. Psychiatric ARs were drug-specific, with anxiety predominating for Ciprofloxacin and Moxifloxacin, and insomnia for Levofloxacin. All major signals were statistically robust (IC025 > 0), confirming distinct compound-specific neuropsychiatric risk profiles. **Conclusions**: The large-scale 16-year analysis demonstrates distinct, drug-specific neuropsychiatric risk profiles. The available evidence supports a non-interchangeable safety profile among FQNs and emphasises the importance of drug-specific risk–benefit assessment in clinical practice.

## 1. Introduction

### 1.1. General Information About Fluoroquinolones

Antibacterial fluoroquinolones (FQNs) are synthetic compounds with antimicrobial activity, whose development was initiated following the discovery of nalidixic acid in 1960 [1,2]. According to the U.S. Food and Drug Administration (FDA), FQNs are indicated for the treatment of a wide range of infectious diseases, including urinary tract infections (UTIs), pyelonephritis, sexually transmitted infections, bacterial prostatitis, intra-abdominal and gastrointestinal infections, skin and soft tissue infections, as well as both community-acquired and hospital-acquired pneumonia. Additionally, Levofloxacin and Moxifloxacin are used off-label in the management of multidrug-resistant (MDR) tuberculosis. FQNs are regarded as versatile antibacterial agents and are available in oral, parenteral, and topical formulations [3,4].

As a result of extensive research into the relationship between chemical structure and biological activity, as well as the growing prevalence of MDR bacteria and diverse infectious diseases, researchers have focused on developing new FQN compounds. The newer agents exhibit enhanced potency, a broader spectrum of antimicrobial activity, and improved pharmacokinetic properties, including superior absorption and tissue distribution [2,5]. Currently, third- and fourth-generation FQNs are utilised in clinical practice due to their favourable pharmacokinetic profiles and broad-spectrum antimicrobial activity. However, FQNs are associated with a range of significant adverse reactions (ARs), which limit their suitability as first-line agents for mild-to-moderate infections. ARs may manifest during treatment or following its discontinuation and can vary in severity and duration, ranging from acute to persistent, and from mild to severe. Also, ARs may affect a single organ system or involve multiple systems simultaneously. Notably, FQNs have been linked to serious and potentially irreversible ARs involving tendons, muscles, joints, peripheral nerves, and the central nervous system (CNS), which may co-occur in the same patient [1,6,7,8,9]. In 2018, the FDA updated the existing warnings in the prescribing information for FQNs to highlight the risk of severe drops in blood sugar levels and potential mental health side effects [7]. In addition, the FDA has warned that FQNs may increase the risk of rare but serious aortic adverse events. Reported complications include aortic dissection and rupture of aortic aneurysms, conditions that may result in life-threatening haemorrhage or death. Such adverse outcomes have primarily been associated with systemic administration of FQNs [10].

### 1.2. FQNs for Systemic Use

The systemic exposure associated with FQNs highlights their potential to affect the central and peripheral nervous systems (PNSs), making neuropsychiatric safety a clinically relevant concern. In current clinical practice, several FQNs from the second (Ciprofloxacin, Ofloxacin, Norfloxacin, Pefloxacin), third (Levofloxacin), and fourth (Delafloxacin, Moxifloxacin) generations are systemically administered (**Table 1**). Several have been withdrawn from the market due to severe ARs. Examples include Grepafloxacin (cardiotoxicity), Temafloxacin (autoimmune haemolytic anaemia), Trovafloxacin (hepatotoxicity), and both Lomefloxacin and Sparfloxacin (phototoxicity and QT interval prolongation) [6,11,12].

### 1.3. Neuropsychiatric ARs Associated with FQNs

Numerous studies on the neuropsychiatric toxicity of FQNs on the CNS and PNS have examined possible mechanisms of toxicity. Still, many of the associated ARs have unknown mechanisms [32]. CNS and PNS side effects associated with FQNs are more commonly reported than those of other systemic antibiotics. In addition, the third- and fourth-generation FQNs (e.g., Levofloxacin and Moxifloxacin) have been associated with an increased risk of neurological or psychiatric ARs compared with second-generation FQNs. ARs to these antibiotics occur in 1% to 4.4% of patients and range from mild (confusion, irritability, insomnia, headache) to severe (convulsions, encephalopathy, depression, suicidal ideation, and psychosis) [33].

Despite accumulating case reports and regulatory warnings, comparative, large-scale evaluations of neuropsychiatric ARs across individual FQNs in settings remain limited. Some commonly reported neuropsychiatric ARs are discussed below, with a focus on their association with FQNs.

*Depression:* In 2023, the UK Medicines and Healthcare Products Regulatory Agency (MHRA) issued a safety advisory warning that FQNs, including Delafloxacin, are associated with an increased risk of serious psychiatric ARs (such as depression, psychosis, and suicidal ideation) and advised clinicians to exercise caution when prescribing these agents, particularly in patients with pre-existing mental health disorders, while ensuring that patients and caregivers are informed of the potential neuropsychiatric risks [34]. However, what remains insufficiently characterised is the comparative magnitude and specificity of these neuropsychiatric risks among individual FQNs, as well as their distribution across different psychiatric outcomes in populations, highlighting the need for large-scale, drug-specific pharmacovigilance analyses.

*Hallucinations:* Clinical evidence suggests a recurrent pattern of acute hallucinations associated with Moxifloxacin, often emerging shortly after treatment initiation and indicating a rapid CNS effect rather than isolated idiosyncratic reactions [35]. The proposed mechanisms consistently implicate FQN-related disruption of inhibitory gamma-aminobutyric acid (GABA)-mediated signalling, enhancement of excitatory *N*-methyl-*D*-aspartate (NMDA)-mediated pathways, and metabolic disturbances such as dysglycaemia, which together may lower the threshold for neuropsychiatric symptoms. The relative contribution of these mechanisms and the extent to which hallucination risk varies between individual FQNs remain insufficiently defined.

*Peripheral neuropathy*: Pharmacovigilance data and epidemiological studies consistently highlight peripheral neuropathy as one of the most frequently reported neurological ARs associated with FQNs, with evidence suggesting that the risk may persist for months after treatment discontinuation, pointing to a potential sustained neurotoxic effect [36]. The association has been observed across multiple agents within the class, including newer-generation FQNs [37], although reported incidence in clinical trials remains low. For other FQNs, there are limited clinical trials available concerning peripheral neuropathy [6]. The biological mechanisms, the duration and reversibility of nerve injury, and the extent of variability in peripheral neuropathy risk among individual FQNs remain insufficiently characterised, largely due to limited prospective data and reliance on spontaneous reporting systems.

*Psychosis*: Available clinical, regulatory, and pharmacovigilance evidence indicates that psychosis and related perceptual disturbances (visual, auditory, or tactile hallucinations) represent a recurrent neuropsychiatric safety signal associated with FQNs, reported across multiple agents including Ciprofloxacin, Levofloxacin, and Delafloxacin [38,39,40]. The associated reactions often present acutely during treatment, may occur from the initial doses, and typically resolve after drug discontinuation, supporting a drug-related and reversible CNS effect. Regulatory product information for Baxdela (Delafloxacin) further highlights a class-wide liability for severe psychiatric ARs, encompassing psychosis, hallucinations, delirium, mood disturbances, and suicidal ideation [41,42]. The comparative frequency, severity, and risk determinants of FQN-associated psychosis across individual compounds remain insufficiently characterised, and the mechanisms sustaining differential susceptibility among patients are still poorly understood, highlighting the need for large-scale, comparative pharmacovigilance studies.

*Seizure potential:* Existing evidence indicates that seizures represent an infrequent but clinically significant neuropsychiatric AR associated with FQNs, typically occurring in the presence of predisposing factors such as prior neurological disease, metabolic disturbances, or impaired renal or hepatic function [43,44,45]. Reported seizure phenotypes include generalised tonic–clonic, confusional, and myoclonic seizures, suggesting a non-specific lowering of the seizure threshold rather than a single uniform mechanism [39,46]. Although seizures have been documented across different FQNs, including newer agents, available data suggest a low absolute incidence, largely derived from isolated cases and early-phase trials [35,39]. Consequently, the true magnitude of seizure risk, its dose-dependence, and potential differences among individual FQNs remain poorly defined, highlighting the need for large-scale pharmacovigilance analyses to better characterise this safety signal in populations.

### 1.4. Rationale for a Pharmacovigilance-Based Assessment of Neuropsychiatric Risk

Currently, the neuropsychiatric safety profile of FQNs remains incompletely characterised at the population level. Existing evidence is largely derived from isolated case reports, small observational studies, or class-level analyses, which limits the ability to distinguish compound-specific risk patterns from class-wide effects. In particular, comparative data evaluating neurological and psychiatric ARs across individual, widely prescribed FQNs in clinical practice are scarce. Moreover, many neuropsychiatric manifestations (ranging from peripheral neuropathy and seizures to psychosis, anxiety, and suicidal ideation) are likely under-recognised and under-reported in clinical practice.

Ciprofloxacin, Levofloxacin, and Moxifloxacin were selected for analysis because they represent widely used FQNs from different generations, with substantial global exposure and longstanding regulatory concern regarding central and PNS toxicity. The FDA FAERS was chosen as the data source due to its ability to enable large-scale evaluation of rare and serious ARs in heterogeneous populations that are not adequately represented in clinical trials. A specific focus on neuropsychiatric ARs was adopted in response to persistent regulatory signals and clinical observations, suggesting that these ARs constitute a clinically pointed and potentially disabling component of FQN toxicity, yet remain insufficiently quantified at the individual-drug level.

### 1.5. Objectives of the Study

The primary objective of this study was to comprehensively characterise and compare the neuropsychiatric safety profiles of three widely used systemic FQNs (Ciprofloxacin, Levofloxacin, and Moxifloxacin) using data from the FAERS database [47].

The secondary objectives were to (i) identify and quantify associations between individual FQNs and specific neurological and psychiatric ARs through disproportionality analyses employing the Information Component (IC), Proportional Reporting Ratio (PRR), and Reporting Odds Ratio (ROR); (ii) distinguish class-related neuropsychiatric effects from compound-specific risk patterns; (iii) examine reporting trends and demographic characteristics associated with neuropsychiatric ARs; and (iv) contextualise pharmacovigilance signals through comparison with published clinical evidence and regulatory safety communications.

Collectively, the objectives of the study aim to support a more nuanced understanding of FQN-associated neuropsychiatric risks and to inform safer, evidence-based prescribing and pharmacovigilance practices.

## 2. Results

A multi-level analytical framework comprising five components was applied to the results: (1) reporting trend by year of neuropsychiatric ARs, (2) demographic characterisation of affected patients, (3) descriptive frequencies of neurological and psychiatric adverse reactions (ARs), (4) disproportionality analysis using the IC, PRR, and ROR to identify significant drug-event associations, and (5) seriousness classification for all AR reports submitted to the FAERS database. All analyses allow quantitative profiling and detection of potential safety signals.

**Figure 1:**
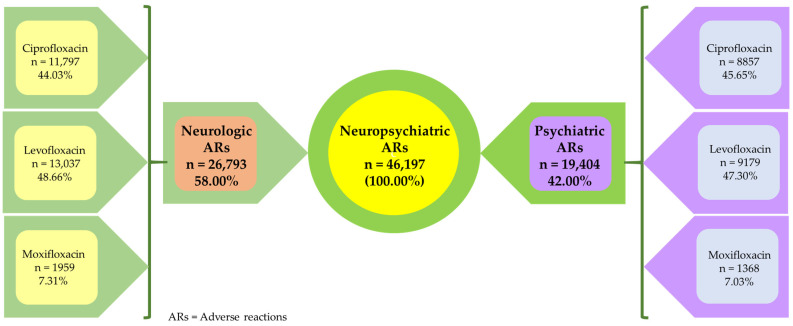
Distribution of neuropsychiatric adverse reactions (ARs) associated with fluoroquinolones (FQNs) reported in the FDA Adverse Event Reporting System (FAERS), 2010–2025. The total number of neuropsychiatric ARs (n = 46,197; 100%) is subdivided into neurologic (n = 26,793; 58.00%) and psychiatric (n = 19,404; 42.00%) categories, with corresponding counts and percentages for Ciprofloxacin, Levofloxacin, and Moxifloxacin (n = number of reports).

Neuropsychiatric ARs were selected using the Peripheral Neuropathy Standardised MedDRA Query (SMQ), as it encompasses a broad spectrum of nervous system-related manifestations, including symptoms with cognitive and psychiatric relevance that may reflect central or peripheral neurotoxicity in spontaneous reporting databases [48]. Based on this SMQ, ARs were further categorised into neurological and psychiatric groups for analysis. The neurological ARs comprised dizziness, somnolence, headache, hypoesthesia, memory impairment, neuralgia, paraesthesia, peripheral neuropathy, seizures, tonic–clonic seizures, and tremor. The psychiatric ARs included agitation, anxiety, confusional state, delirium, depression, hallucinations, insomnia, panic attack, psychotic disorders, sleep disorder, and suicidal ideation. A comparative analysis was conducted on 95,968 individual case safety reports, including 46,197 neuropsychiatric ARs. The reports were associated with three FQNs: Ciprofloxacin (second generation), Levofloxacin (third generation), and Moxifloxacin (fourth generation) (**Figure 1**).

### 2.1. Reporting Trend by Year of Neuropsychiatric ARs

**Figure 2:**
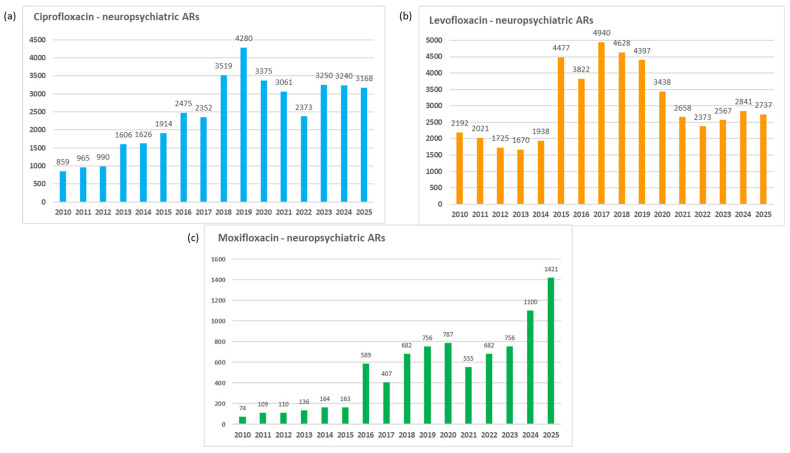
Annual trends in reported neuropsychiatric adverse reactions (ARs) from 2010 to 2025 for individual fluoroquinolones (FQNs): (**a**) Ciprofloxacin, (**b**) Levofloxacin, and (**c**) Moxifloxacin. The bar charts illustrate year-by-year fluctuations in the number of reported cases for each drug.

A detailed examination of the yearly reporting trends highlights distinct neuropsychiatric AR profiles for each of the three FQNs; therefore, individual summary trends are presented below. Neuropsychiatric ARs associated with Ciprofloxacin showed a moderate but steady upward trend between 2010 and 2025 (**Figure 2a**). Levofloxacin exhibited a markedly higher reporting impact and more pronounced fluctuations than Ciprofloxacin (**Figure 2b**). Moxifloxacin had the lowest absolute number of neuropsychiatric AR reports but showed the steepest relative increase over time (**Figure 2c**).

### 2.2. General Characteristics of Reports

An assessment of the general characteristics of the reports was conducted to describe the demographic profile and reporting structure of neuropsychiatric ARs associated with the selected FQNs.

#### 2.2.1. Demographics of Neuropsychiatric ARs

Demographic characteristics, including sex and age, were analysed to identify differences in the distribution of neuropsychiatric ARs among patients exposed to the three FQNs.

**Figure 3:**
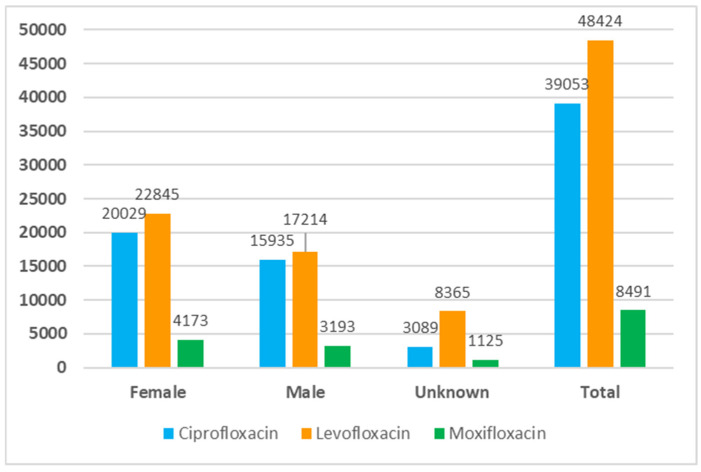
Distribution of neuropsychiatric AR reports by sex for Ciprofloxacin, Levofloxacin, and Moxifloxacin. The figure depicts the relative contribution of female, male, and unknown-sex cases to the total number of reports for each FQN.

*Sex distribution*: Demographic analysis of neuropsychiatric ARs associated with the three selected FQNs revealed that 47,047 (49.02%) reports involved female patients, 36,342 (37.87%) involved male patients, and 12,579 (13.11%) involved patients of unspecified gender. Data on the sex distribution of total neuropsychiatric ARs are represented in **Figure 3**.

**Figure 4:**
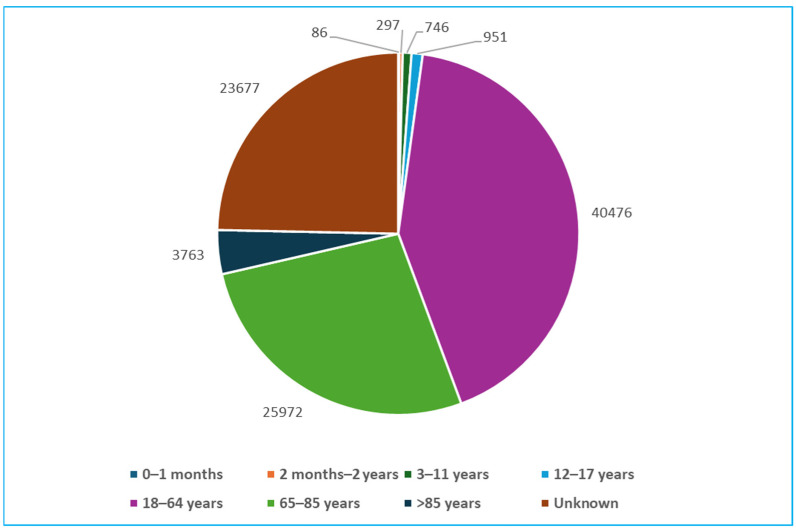
Age distribution of reported neuropsychiatric adverse reactions (ARs) associated with Ciprofloxacin, Levofloxacin, and Moxifloxacin in the FAERS database (2010–2025). The absolute numbers of reports are shown for each age group, including cases with unknown age.

*Age distribution:* Analysis of the age distribution of neuropsychiatric AR reports revealed that cases were predominantly reported among adults aged 18–64 years, who accounted for the largest share across all three FQNs (**Figure 4**). Individuals aged 18–64 years accounted for 44.77% of Ciprofloxacin, 45.33% of Levofloxacin, and 9.90% of Moxifloxacin adverse-event reports, indicating that middle-aged adults represented the largest proportion of reported exposures for Ciprofloxacin and Levofloxacin. In contrast, Moxifloxacin reports were less frequent in this age group (**Table 2**). Older adults aged 65–85 years also contributed substantially, especially for Ciprofloxacin and Levofloxacin, whereas reports in paediatric age groups remained comparatively low.

#### 2.2.2. Descriptive Analysis of Neurological ARs

A descriptive analysis of neurological ARs was performed using 26,793 reports documenting CNS pathologies associated with Ciprofloxacin, Levofloxacin and Moxifloxacin. **Table 3** presents the number and the proportion of reported ARs. Levofloxacin and Moxifloxacin showed a disproportionate concentration of peripheral neuropathy reports, while Ciprofloxacin predominantly generated central symptoms such as headache and dizziness.

The most frequently reported neurological ARs during the targeted period were headache with Ciprofloxacin (1991 reports, 16.88%), peripheral neuropathy with Levofloxacin (2945 reports, 22.59%) and Moxifloxacin (476 reports, 24.30%). Peripheral neuropathy accounts for the highest number of reports (4856, 18.12% of total neurological reports).

**Figure 5:**
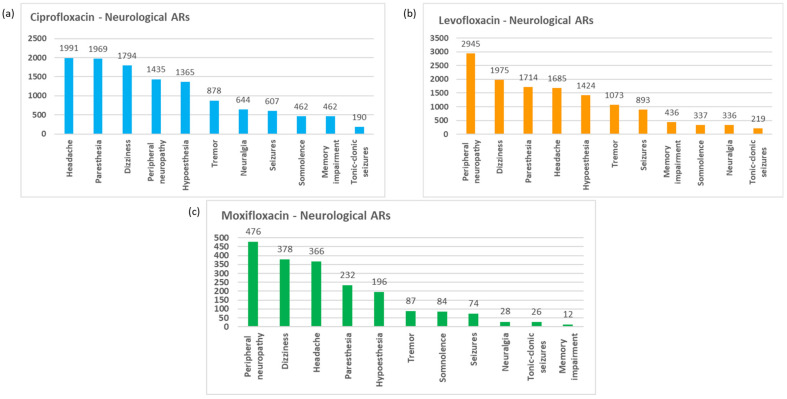
Neurological adverse reactions (ARs) reported in association with (**a**) Ciprofloxacin, (**b**) Levofloxacin, and (**c**) Moxifloxacin. The figure displays the distribution and absolute number of neurological ARs, highlighting the most frequently reported events during the 2010–2025 period.

*Ciprofloxacin:* Among the neurological ARs reported for Ciprofloxacin, headache accounted for the highest number of cases, totalling 1991 (16.9% of all neurological ARs associated with this drug) (**Figure 5a**). Headache was closely followed by paraesthesia (1969 reports; 16.7%) and dizziness (1794 reports; 15.2%), indicating that the most frequently encountered neurological manifestations were predominantly non-severe but highly prevalent symptoms.

*Levofloxacin:* Peripheral neuropathy constituted the neurological manifestation with the highest number of reports, totalling 2945 cases (22.59% of all neurological ARs associated with this drug) (**Figure 5b**). Other commonly reported neurological reactions, such as dizziness (1975 reports; 15.15%) and paraesthesia (1714 reports; 13.15%), occurred at notably lower frequencies.

*Moxifloxacin*: Peripheral neuropathy was the neurological event with the highest number of reports, totalling 476 cases (24.30% of all attributed neurological ARs) (**Figure 5c**); this made it the most prominent neurological safety concern associated with Moxifloxacin, exceeding other commonly reported reactions such as dizziness (378 reports; 19.30%), headache (366 reports; 18.68%), and paraesthesia (232 reports; 11.84%).

#### 2.2.3. Descriptive Analysis of Psychiatric ARs

A descriptive analysis of psychiatric ARs was performed using 19,404 reports documenting ARs associated with Ciprofloxacin, Levofloxacin and Moxifloxacin. **Table 4** presents the number and the proportion of reported ARs. Anxiety dominated psychiatric reports for Ciprofloxacin and Moxifloxacin, whereas insomnia was uniquely prominent for Levofloxacin, revealing a drug-specific psychiatric signature.

The most frequently reported psychiatric ARs during the targeted period were anxiety for Ciprofloxacin (1971 reports, 22.25%) and Moxifloxacin (364 reports, 26.61%), and insomnia for Levofloxacin (2016 reports, 21,96%). Anxiety accounted for the highest number of reports (4133, 21.30% of total psychiatric reports) for the three selected FQNs.

**Figure 6:**
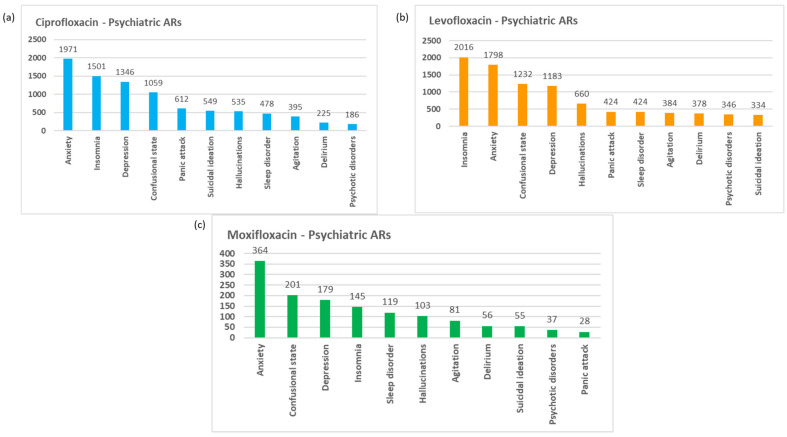
Psychiatric adverse reactions (ARs) reported in association with (**a**) Ciprofloxacin, (**b**) Levofloxacin, and (**c**) Moxifloxacin. The figure illustrates the distribution and absolute number of psychiatric ARs, highlighting the most frequently reported events during the 2010–2025 period.

*Ciprofloxacin:* Anxiety is the predominant psychiatric manifestation in the Ciprofloxacin safety profile; it was the most frequently reported psychiatric AR, with 1971 cases (22.25% of all psychiatric ARs associated with this agent) (**Figure 6a**). Other commonly reported reactions, such as insomnia (1501 reports; 16.95%), depression (1346 reports; 15.20%), and confusional state (1059 reports; 11.96%), occurred at lower but still notable frequencies.

*Levofloxacin:* Insomnia was the most frequently reported psychiatric AR, with 2016 cases, representing 21.96% of all attributed psychiatric ARs (**Figure 6b**), which made it the predominant psychiatric manifestation in the Levofloxacin safety profile. Other commonly reported psychiatric ARs included anxiety (1798 reports; 19.59%) and a confusional state (1232 reports; 13.42%), while more severe manifestations such as psychotic disorders or suicidal ideation occurred at much lower frequencies.

*Moxifloxacin*: Anxiety represented the most frequently reported psychiatric AR, with 364 cases, accounting for 26.61% of all associated psychiatric ARs (**Figure 6c**). Similarly to Ciprofloxacin, anxiety is the predominant psychiatric manifestation linked to Moxifloxacin. Other frequently reported reactions included confusional state (201 reports; 14.69%), depression (179 reports; 13.08%) and insomnia (145 reports; 10.60%), although these occurred at substantially lower proportions.

### 2.3. Disproportionality Analysis

To evaluate the strength of association between each FQN and the reported neuropsychiatric ARs, a disproportionality analysis was performed using IC, PRR, and ROR. In this context, IC025 represents the lower bound of the 95% credibility interval for the IC; values > 0 indicate a statistically significant and stable association [49,50,51,52]. Similarly, PRR025, the lower bound of the 95% confidence interval for the PRR, supports the presence of a significant disproportionality when >1. The ROR quantifies the odds of reporting a reaction for a specific drug relative to all other drugs, with values > 1 indicating a potential safety signal and higher values reflecting stronger associations. The complementary measures collectively provide a robust framework for identifying pointed pharmacovigilance signals across the dataset [50,53,54].

#### 2.3.1. Ciprofloxacin

The disproportionality analysis identified several neuropsychiatric ARs with strong statistical associations with Ciprofloxacin, reflected by consistently elevated IC, PRR, and ROR values (**Table 5**). The most prominent signals were observed for neuralgia, panic attacks, peripheral neuropathy, and paraesthesia, which demonstrated the highest disproportionality metrics across all evaluated ARs. Neuralgia (IC 4.39; ROR 21.90) and peripheral neuropathy (IC 3.59; ROR 12.67) demonstrated particularly strong associations. Overall, the analysis suggests that Ciprofloxacin exhibits a notable neuropsychiatric risk profile, especially for ARs involving sensory disturbances and peripheral nerve dysfunction.

#### 2.3.2. Levofloxacin

Analysis of disproportionality metrics for Levofloxacin revealed a distinct cluster of neuropsychiatric ARs with elevated IC, PRR, and ROR values (**Table 6**). Peripheral neuropathy represented the most pronounced signal (IC 4.32; ROR 21.12), highlighting a strong association with Levofloxacin use. Several other events, including neuralgia (IC 3.14; ROR 9.01), delirium (IC 2.92; ROR 7.71), paraesthesia (IC 2.92; ROR 7.71), and confusional state (IC 2.40; ROR 5.35), demonstrated considerable disproportionality, each with IC values exceeding 2.3. These consistent and markedly elevated values highlight Levofloxacin’s distinct neuropsychiatric risk profile, particularly in relation to peripheral nerve involvement and acute cognitive disturbances.

#### 2.3.3. Moxifloxacin

The disproportionality analysis for Moxifloxacin showed several neuropsychiatric ARs with statistically significant associations, as indicated by elevated IC, PRR, and ROR values (**Table 7**). The strongest safety signal was observed for peripheral neuropathy, which demonstrated markedly high disproportionality metrics (IC 4.20; ROR 19.55), highlighting a robust link between Moxifloxacin and peripheral nerve toxicity. Additional reactions, such as delirium (IC 2.68; ROR 6.44), paraesthesia (IC 2.55; ROR 5.99), hallucinations (IC 2.44; ROR 5.49), and confusional state (IC 2.30; ROR 5.01), also exhibited significant disproportionality values. Although the overall reporting volume for Moxifloxacin is lower than for the other two FQNs, the consistency of these elevated metrics highlights pointed neuropsychiatric safety signals associated with this FQN.

To assess the robustness of the identified disproportionality signals, the stability of the results was evaluated by examining the lower bounds of the IC025 and the concordance between IC and ROR estimates. All major neuropsychiatric signals retained IC025 values above zero and showed consistent IC–ROR patterns across the three FQNs, indicating robust and stable associations that were unlikely to result from reporting artefacts.

### 2.4. Seriousness Classification for All AR Reports Submitted to the FAERS Database

**Figure 7:**
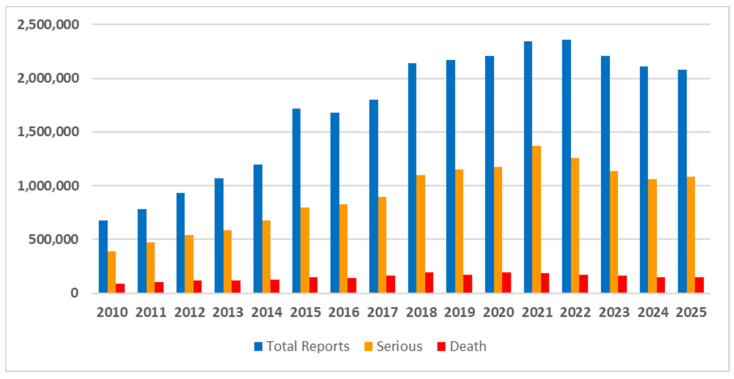
Seriousness classification of all AR reports submitted to the FAERS database across the 2010–2025 period. The figure displays the annual numbers of total reports, serious cases, and deaths, illustrating the overall reporting impact and the proportion of severe clinical events captured in the database.

Since FAERS includes all drug classes, the seriousness classification is presented to contextualise the proportion of severe and fatal reports within the entire database rather than among FQNs specifically. The FAERS dataset reflects the seriousness classification for all AR reports submitted to the database by year (**Figure 7**). The values for “Serious” and “Death” correspond to the totality of reported cases across all therapeutic areas and drug classes, representing the global impact of severe outcomes captured by the system.

**Figure 8:**
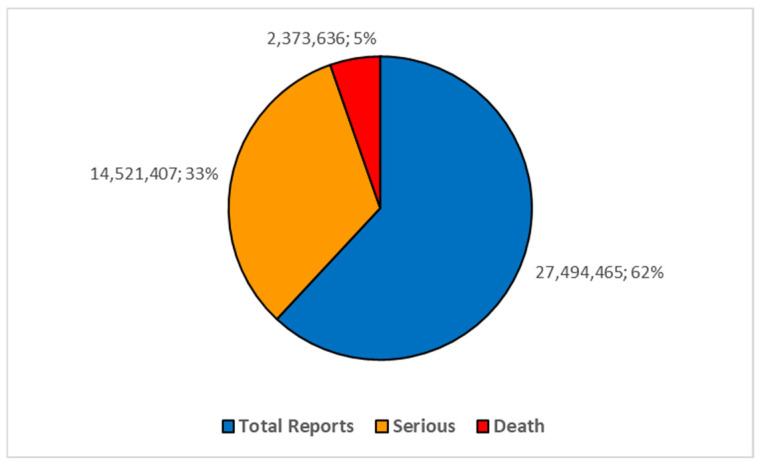
Overall distribution of adverse reaction (AR) reports in the FAERS database (2010–2025), showing total reports alongside the proportions classified as serious and fatal cases, highlighting the overall burden of severe clinical outcomes.

The distribution shows that serious events account for a substantial share of all submissions, with fatal cases representing a smaller but notable portion of the overall reporting burden (**Figure 8**).

Overall, the results are strengthened by the large-scale FAERS dataset and the consistent identification of robust, statistically significant neuropsychiatric signals across all three FQNs. The integration of descriptive analyses with multiple disproportionality methods (IC, PRR, ROR) enabled clear differentiation of compound-specific risk profiles, highlighting both shared class effects and distinct neuropsychiatric patterns for each drug.

## 3. Discussion

The present pharmacovigilance analysis demonstrates that neuropsychiatric ARs associated with FQNs are not uniform class effects but exhibit distinct, compound-specific profiles. Peripheral neuropathy emerged as the most consistent and strongest signal for Levofloxacin and Moxifloxacin, whereas Ciprofloxacin was more strongly associated with centrally mediated symptoms. The results provide quantitative evidence supporting drug-specific risk stratification in clinical practice.

### 3.1. General Interpretation of the Main Results

The study provides a large-scale evaluation of neuropsychiatric safety across three FQNs using FAERS data (2010–2025), demonstrating distinct, drug-specific risk profiles. Neuropsychiatric ARs were consistently reported and varied in frequency, type, and strength of association, reflecting underlying pharmacological, demographic, and clinical patterns. The three FQNs showed distinct and drug-specific reporting trajectories for neuropsychiatric ARs, which are discussed separately.

Ciprofloxacin showed a gradual but consistent increase, marked by two peaks (2016 and 2019), followed by stabilisation at high reporting levels, indicating a sustained impact of neuropsychiatric complaints over time (**Figure 2a**). The number of reports increased from 859 in 2010 to a first peak in 2016 (2475), then rose again starting in 2018, reaching a high in 2019 (4280). After 2020, yearly reports stabilised between 2373 and 3250 cases, indicating a sustained and consistently high volume of neuropsychiatric ARs reported for Ciprofloxacin across the entire study period.

Levofloxacin demonstrated the highest absolute number of reports and the greatest year-to-year variability, with a pronounced spike in 2017 and persistently elevated reporting thereafter, reflecting both its wide clinical use and a strong neuropsychiatric signal in clinical practice (**Figure 2b**). Neuropsychiatric AR reports increased from 2192 in 2010 to a substantial peak in 2017 (4940 reports), followed by persistently high annual counts through 2025. Although a slight decrease occurred between 2020 and 2022, reports rose again afterwards, remaining above 2500 per year. Overall, Levofloxacin shows the highest and most variable neuropsychiatric AR reporting trend.

In contrast, Moxifloxacin had the lowest total volume but showed the steepest proportional rise, especially after 2023, culminating in a sharp increase from 2024 to 2025 (**Figure 2c**). Reports rose from 74 cases in 2010 to 589 in 2016, then continued to grow to 682–756 cases between 2018 and 2023. The last two years saw a sharp escalation, with reports rising to 1100 in 2024 and 1421 in 2025, indicating a rapid increase in Moxifloxacin reporting.

The cumulative evidence shows that neuropsychiatric manifestations constitute a distinct component of the AR profiles associated with Ciprofloxacin, Levofloxacin, and Moxifloxacin (Section 2.1). The distinct patterns observed among the three FQNs highlight the importance of drug-specific risk assessment, particularly for vulnerable populations such as older adults or individuals receiving concomitant CNS-active medication. The obtained data support the need for continued surveillance, enhanced prescriber awareness, and more targeted studies to elucidate the mechanisms responsible for neuropsychiatric ARs.

### 3.2. Comparison with Previously Published Data

The patterns identified in this study are consistent with growing evidence documenting the neuropsychiatric risks associated with FQN therapy. Prior pharmacovigilance research has demonstrated that psychiatric ARs constitute a substantial proportion of FQN-associated safety signals. A large FAERS-based analysis (2004–2023) conducted by Xie W.-L. et al. (2024) [55] identified more than 27,000 psychiatric ARs linked to FQNs, with mood disorders (particularly anxiety, depression, and delirium) representing the most frequently reported manifestations. Ciprofloxacin showed stronger associations with depression and suicidal ideation, while Moxifloxacin was more frequently implicated in delirium. Such observations are congruent with the FQN-specific psychiatric signatures detected in our study, thereby affirming that each FQN manifests a uniquely patterned neuropsychiatric risk profile within the broader class. Our results showed strong disproportionality signals for anxiety, insomnia, delirium, hallucinations, paraesthesia, and peripheral neuropathy (Section 2.3), which align with the analysis by Xie W.-L. et al. (2024). Notably, our data confirm stronger associations for peripheral neuropathy with Levofloxacin (**Table 6**) and Moxifloxacin (**Table 7**), consistent with their observations.

Wierzbiński P. et al. (2023) [38] published a systematic review which further supports these observations, reporting that psychiatric ARs such as anxiety, insomnia, psychosis, and depressive symptoms occur in approximately 1–4.4% of patients receiving FQNs. Hypotheses regarding the mechanism of psychiatric ARs focus on GABA_A_ receptor antagonism and NMDA receptor overactivation; these mechanisms align with the high frequency of CNS excitatory symptoms detected in the current analysis. The review emphasises that susceptibility is higher in older adults and in those with polypharmacy. Our disproportionality analysis supports the existing evidence and further highlights anxiety as a psychiatric AR for Ciprofloxacin (**Figure 6a**) and Moxifloxacin (**Figure 6c**). At the same time, insomnia was the most reported psychiatric AR for Levofloxacin (**Figure 6b**), supporting drug-specific neuropsychiatric patterns.

Abusafiyah N. et al. (2023) [56] conducted a narrative review and identified FQNs as the antibiotic class with the highest rate of severe psychiatric effects (anxiety, confusion, agitation, and hallucinations). The review included a clinical case in which Moxifloxacin prophylaxis caused pronounced anxiety and confusion within 48 h, and noted comparable effects across the class, including Levofloxacin. Our data corroborate this pattern, particularly the elevated IC and ROR values for hallucinations and confusional state, which were especially pronounced for Ciprofloxacin (**Table 5**) and Moxifloxacin (**Table 7**).

A large retrospective cohort comparing FQNs (including Levofloxacin and Moxifloxacin) with macrolides (conducted by Gueta I. et al. in 2024 [57]) found a low but measurable seizure risk. Although the overall incidence was low (1:5422 treatment days for FQN), causality was “probable” or “possible” in many cases, based on the Naranjo Adverse Drug Reaction Probability Scale. In our study, the obtained results identified seizures as a significant but less frequent AR, consistent with the 2024 cohort study by Gueta I. et al. (2024), which found a measurable but low seizure incidence among FQN users. Our IC and ROR values indicate a higher association with seizures for Levofloxacin (IC 1.87; ROR 3.69) than for Moxifloxacin (IC 0.79; ROR 1.74), mirroring their observations.

The evidence is also consistent with population-based pharmacoepidemiological studies. Zhang Y. et al. (2024) [58] conducted a self-controlled case series; they reported a significant increase in acute neuropsychiatric events during FQN exposure (Incidence Rate Ratio (IRR) = 2.11), with the elevated risk persisting briefly after treatment cessation. Thus, patients were more than twice as likely to experience an acute neuropsychiatric event while taking FQNs compared with their own baseline periods. Psychotic symptoms and cognitive disturbances followed similar temporal patterns. Notably, Levofloxacin and Ciprofloxacin accounted for the majority of prescriptions (46% and 41%, respectively), suggesting that higher utilisation may partially explain their stronger signal intensity in datasets. The results of this study align with our disproportionality analysis, in which both FQNs demonstrated marked reporting enrichment for psychiatric ARs such as hallucinations, confusion, and agitation. Similarly, our results revealed high disproportionality signals for acute neuropsychiatric symptoms, particularly agitation, confusion, and hallucinations (Section 2.3), indicating that these ARs are likely to occur early during treatment.

Literature focused specifically on neurotoxicity further corroborates our observations. A review by Kamath A. (2013) focused on FQN-induced CNS effects concluded that Ciprofloxacin and Ofloxacin carry the greatest propensity for CNS toxicity. Levofloxacin also produces significant neuropsychiatric events when risk factors are present (e.g., advanced age, renal impairment, neurological comorbidities). The review highlights that these reactions frequently arise from reversible neurochemical disturbances but remain under-recognised in clinical practice, consistent with the recurrent under-reporting observed across pharmacovigilance systems [59]. Our data confirm that Ciprofloxacin shows a strong association with neuralgia, paraesthesia, and hallucinations (**Table 4**), and highlight its prominent CNS risk profile within the FQNs class.

Recent evidence has expanded the spectrum of recognised FQN-related psychiatric effects. A FAERS-based disproportionality analysis conducted by Omrani M.A. et al. (2025) demonstrated a 6- to 10-fold increase in nightmare reporting for Ciprofloxacin, Levofloxacin, and Moxifloxacin compared with other commonly prescribed antibiotics. The study suggests that neuropsychiatric toxicity encompasses a broader range of ARs than traditionally appreciated, extending beyond more commonly investigated endpoints such as anxiety and psychosis [60]. Although nightmares were not among the most frequently reported events in our dataset, they remain relevant for illustrating the breadth and variability of FQN-associated neuropsychiatric ARs. Furthermore, our results diverge from those of Omrani M.A. et al. (2025). Our findings suggest that while this symptom represents a recognised component of the FQN neuropsychiatric spectrum, its reporting frequency may vary considerably across populations, methodologies, or exposure contexts. Therefore, FQN-associated neurotoxicity encompasses a broader range of psychiatric manifestations than those most prominently detected in our disproportionality analysis.

The published evidence provides robust support for the differentiated neuropsychiatric risk profiles identified in the present study. The concordance between prior research and our results highlights the necessity of employing a nuanced, drug-specific approach to safety evaluation, particularly in patient subgroups with heightened susceptibility to CNS-related ARs. Collectively, these considerations highlight the critical importance of increased clinical vigilance and individualised antimicrobial management when selecting an antibiotic from among the three targeted FQNs.

### 3.3. Evidence from Published Case Reports of FQN-Induced Neuropsychiatric ARs

Presented below are representative cases demonstrating how both Levofloxacin and Moxifloxacin can precipitate sudden-onset psychotic and dissociative symptoms, accentuating the need to improve clinicians’ awareness of this under-recognized AR profile.

Kandasamy A. et al. (2012) [39] describe three case reports of acute anxiety and insomnia associated with Levofloxacin use, highlighting a rare but significant neuropsychiatric adverse effect of FQNs. All three patients developed prominent anxiety symptoms, restlessness, and sleep disturbance shortly after initiation of Levofloxacin, despite having no prior history of psychiatric illness or other identifiable causes, and symptoms resolved promptly after discontinuation of the drug. The authors discuss possible mechanisms, including FQN-related GABA antagonism and CNS excitation. Another published case report described a previously healthy adult male who developed acute psychosis after only three doses of Moxifloxacin, manifesting hallucinations, anxiety, insomnia, and agitation despite the absence of any psychiatric history or identifiable predisposing factors. The neuropsychiatric symptoms emerged rapidly and resolved completely within approximately 12 h of discontinuing the antibiotic, a temporal pattern that strongly supports a probable causal association according to the Naranjo Adverse Drug Reaction Probability Scale. It could be affirmed that Moxifloxacin is capable of inducing abrupt and severe neuropsychiatric reactions even in individuals considered to be at low baseline risk [61].

A healthy 22-year-old developed derealization, depersonalization, agitation, auditory hallucinations, insomnia, and nightmares within 3 h of receiving a single orally administered 500 mg dose of Levofloxacin. Symptoms lasted 3 days and fully resolved spontaneously. This demonstrates the extreme rapidity of CNS penetration and highlights the risk even in young adults without comorbidities [62]. Another case report of acute psychotic symptoms occurred after a single dose of Levofloxacin, illustrating the potential for rapid-onset and severe neuropsychiatric ARs associated with FQNs. The patient, who had no prior psychiatric history, developed acute psychosis shortly after Levofloxacin administration, and symptoms resolved following prompt discontinuation of the drug, without evidence of an alternative medical or toxic cause. The authors highlighted possible mechanisms such as FQN-induced CNS excitation, including GABA receptor inhibition [62].

Another case report described a woman receiving intravenous Levofloxacin (750 mg per day) who developed acute visual hallucinations beginning on day 2 of therapy. The patient’s neurologic, endocrine, and toxicology screens were normal, strongly suggesting probable drug-induced psychosis (Naranjo score + 6). Symptoms completely resolved after discontinuation. The authors stress that true prevalence is likely underestimated and that clinicians must consider Levofloxacin as a cause of abrupt psychotic symptoms in otherwise healthy individuals [63]. In addition, another case report presented a Moxifloxacin-induced acute mental status change in an 81-year-old man who developed bizarre behaviour, altered consciousness, and slurred speech shortly after initiation of the antibiotic. The patient had no alternative explanation for the symptoms, which resolved completely after discontinuation of Moxifloxacin, supporting a causal relationship. Although CNS ARs of FQNs are rare (occurring in less than 1% of patients), they can be severe and clinically significant, particularly in elderly individuals [64].

A rare paediatric case described by Getova-Kolarova V. et al. (2025) [65] reported severe insomnia and psychotic manifestations in a toddler following off-label nasal administration of Moxifloxacin ophthalmic solution. The neuropsychiatric symptoms resolved rapidly upon discontinuation of the medication, strongly implicating Moxifloxacin as the causal agent. The heightened vulnerability of the developing paediatric CNS to FQN-induced neurotoxicity was stressed, even when exposure occurs through non-systemic and unconventional routes of administration.

### 3.4. Mechanisms’ Plausibility Described in the Literature

Several mechanisms have been proposed to account for these neuropsychiatric effects. FQNs have been linked to neurotoxic effects primarily through their ability to inhibit GABA receptors, a mechanism which has been reported across multiple studies [11,59,66,67]. FQNs impact the CNS by producing imbalances between excitatory and inhibitory neurotransmitters. The excitatory action on the CNS is due to both inhibition of GABA_A_ receptors, by blocking the physiological binding of GABA to specific receptors, and stimulation of NMDA receptors [32,68,69]. ARs affecting the CNS can be categorised into two types: those arising from direct interactions with neural receptors, and those resulting from pharmacodynamic or pharmacokinetic interactions with other therapeutic agents [68]. The mechanisms of neuropsychiatric toxicity are diverse, including pharmacokinetic or pharmacodynamic interactions with other CNS-acting therapeutic agents or direct CNS action of FQNs [70].

A 2016 investigative report by Kaur K. et al. provides a comprehensive examination of FQN-related neuropsychiatric and mitochondrial toxicity, integrating clinical observations from affected patients, pharmacovigilance signal analyses, and mechanisms from basic scientific research. The authors document long-term neurologic and psychiatric manifestations (including cognitive impairment, mood instability, sensory neuropathies, and persistent neuropsychiatric disturbances) reported by individuals exposed to Ciprofloxacin, Levofloxacin, or Moxifloxacin. It was highlighted that these effects may persist well beyond drug discontinuation, suggesting the presence of mechanisms extending beyond transient neurotransmitter interactions. The report demonstrates that FQNs can impair mitochondrial function, induce oxidative stress, and disrupt cellular homeostasis in neuronal tissues, offering a plausible biological basis for chronic symptomatology [71].

The study of Zhang Y. et al. (2025) [72] employed an integrated bioinformatics and network toxicology approach to investigate the molecular mechanisms underlying Levofloxacin-induced CNS injury. Analysis of differentially expressed genes from public databases identified HSP90AA1 as a central target, supported by protein–protein interaction analysis, ROC validation, and molecular docking and 100 ns molecular dynamics simulations, which demonstrated strong and stable binding between Levofloxacin and HSP90AA1 (−9.9 kcal/mol). Pathway enrichment analysis implicated nucleotide-binding oligomerisation domain (NOD)-like receptor signalling, neutrophil extracellular trap formation, and mitogen-activated protein kinase (MAPK) signalling as key processes. Overall, the results suggest that Levofloxacin may induce CNS injury by disrupting neuronal homeostasis through HSP90AA1-mediated inflammatory pathways, providing support for its reported neurotoxic effects and informing risk mitigation in clinical use.

**Figure 9:**
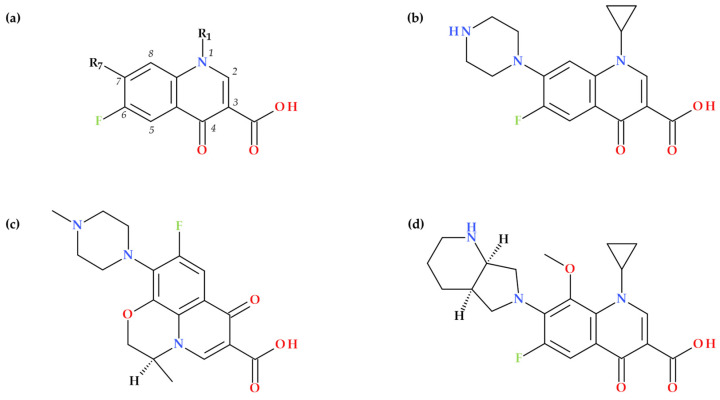
Antibacterial fluoroquinolones (FQNs): (**a**) General chemical structure and numbering of atoms; (**b**) Ciprofloxacin; (**c**) Levofloxacin; (**d**) Moxifloxacin.

At the same time, neuropsychiatric ARs depend on several factors, including the compound’s chemical structure (**Figure 9**), the patient’s physiopathological state (renal function or blood–brain barrier affected), and the polymedication used [69]. Structural features (particularly unsubstituted piperazine or pyrrolidinyl groups at the C7 position) are believed to play a key role in facilitating this interaction with GABAergic pathways. Moreover, FQN derivatives containing unsubstituted heterocyclic moieties at C7 appear more prone to causing CNS ARs, supporting earlier observations of structure-dependent neurotoxicity [6].

Studies investigating the relationship between the chemical structure of FQNs and their neurotoxic potential have demonstrated that substitutions at position C7 are associated with CNS ARs. Specifically, the nature of the substituent at this position influences the risk level, with the following order of increasing neurotoxicity: alkyl < unsubstituted piperazine < unsubstituted pyrrolidinyl < pyrrolidine < piperazine. The CNS-related ARs are closely linked to the binding and inhibition of GABAergic receptors, with piperazine and pyrrolidine moieties playing a significant role in this interaction. Moreover, the presence of unsubstituted piperazine or pyrrolidinyl groups appears to be critical for effective interaction with GABAergic receptors [69,70,73,74].

The presence of methyl-substituted piperazinyl moiety at C7 in Levofloxacin is consistent with structure–activity relationship studies identifying this substituent as a critical determinant of GABAergic receptor inhibition and CNS ARs among FQNs. The chemical structure of Moxifloxacin, characterised by a bulky, substituted C-7 azabicycle (pyrrolidine–piperidine) moiety, could be associated with GABAergic inhibition, which may explain its relatively reduced neurotoxic potential.

Overall, multiple biological pathways may contribute to the neuropsychiatric toxicity associated with FQNs.

### 3.5. Interpretation of Neuropsychiatric Patterns

The pattern suggests that recognition of Moxifloxacin-associated neuropsychiatric risks is growing, despite the medication’s lower overall utilisation. The observed reporting trajectories indicate a progressive intensification of neuropsychiatric AR reporting over time, likely shaped by evolving prescription habits, regulatory communications, and heightened awareness among clinicians and patients. The overall magnitude of reported cases varied considerably among the examined FQNs.

Levofloxacin had the most neuropsychiatric ARs, followed by Ciprofloxacin, whereas Moxifloxacin had considerably fewer reports (Section 2.1). These differences likely reflect variations in prescription, as Ciprofloxacin and Levofloxacin maintain broader prescribing rates globally, while Moxifloxacin use is more restricted [75,76,77]. Although exposure differed, the proportions of specific neuropsychiatric ARs were not uniform across the three FQNs, suggesting that reporting patterns cannot be attributed solely to differences in prescription volume.

#### 3.5.1. Differences Among Demographic Characteristics

Demographic characteristics contribute critically to the contextual understanding of the results.

1. *Sex distribution*

Female patients reported slightly more ARs than males, aligning with known sex-related differences in pharmacovigilance reporting and potentially in drug metabolism (**Figure 3**). Large-scale analyses consistently demonstrate that women submit more AR reports than men [78,79,80].

Several mechanisms have been proposed to explain this disparity. Biological factors, including sex-related differences in pharmacokinetics and pharmacodynamics (such as lower lean body mass, reduced hepatic clearance, and distinct cytochrome P450 activity in women), may increase susceptibility to certain ARs. Behavioural and sociocultural contributors also play a notable role. Women generally exhibit higher healthcare-seeking behaviour, greater engagement with medical systems, and increased medication use overall, which collectively elevate the likelihood of both experiencing and reporting ARs. Additionally, scoping reviews of international pharmacovigilance databases confirm that women are involved in more AR reports than men across regions and drug categories. Within the context of FQNs safety, this imbalance may therefore reflect not only biological vulnerability but also the broader, well-documented trend of more active ARs reporting among women. Such patterns should be considered when interpreting neuropsychiatric signal strength and when developing sex-sensitive risk communication strategies [78,79,81,82,83].

2. *Age distribution*

Adults aged 18–64 years constituted the majority of reported cases, reflecting the age group most frequently exposed to FQNs in clinical practice (**Figure 4**). Older adults also contributed substantially to the neuropsychiatric impact; this was expected, given their higher susceptibility to CNS effects and greater likelihood of polypharmacy.

Across all age categories, Moxifloxacin displayed markedly fewer reports overall, consistent with its lower total reporting volume (**Table 2**). However, its relative distribution followed a pattern similar to that of the other two FQNs, with most reports arising in adult and older adult populations. Only a very small proportion of cases were reported in infants, young children, or adolescents for any of the compounds. Collectively, these results show that neuropsychiatric ARs are reported most frequently in adults, with a secondary peak in older age groups, reflecting both prescribing patterns and population susceptibility. This age-related pattern is consistent with global pharmacovigilance evidence showing that AR reports arise predominantly from adult and older adult populations, with minimal contribution from paediatric age groups, reflecting both prescribing practices and age-dependent susceptibility to ARs [78,84].

The predominance of reports from adults is consistent with current prescribing practices, as FQNs are primarily indicated for infections that occur most frequently in adults. Their broad-spectrum coverage, oral availability, and use in conditions such as urinary tract infections, respiratory tract infections, and skin and soft-tissue infections result in substantially higher exposure among adults compared with paediatric patients [6,85]. Consequently, the higher proportion of neuropsychiatric ARs reported in individuals aged 18–64 years, followed by those 65–85 years, likely reflects the utilisation patterns of these agents rather than an inherent increased susceptibility in these groups.

#### 3.5.2. Neuropsychiatric Risk Characterisation of FQNs

##### Neurological ARs

*Discussions concerning descriptive analysis*

The descriptive analysis of neurological ARs reveals marked heterogeneity in the neurological safety profiles of the three evaluated FQNs. Symptoms such as headache, paraesthesia, and dizziness were most prominently associated with Ciprofloxacin (**Figure 5a**). Thus, Levofloxacin and Moxifloxacin exhibited a disproportionate number of peripheral neuropathy reports (**Figure 5b,c**). The observed divergence suggests that neurological toxicity within the FQN class does not represent a uniform class effect but rather reflects compound-specific patterns, potentially induced by differences in pharmacokinetics, tissue distribution, and structural determinants that influence neuronal affinity [46,70]. The predominance of headache and dizziness in patients taking Ciprofloxacin may indicate transient CNS excitatory effects [43,59]. In contrast, the higher reporting of peripheral neuropathy in conjunction with Levofloxacin and Moxifloxacin administration aligns with growing regulatory and epidemiological concern regarding the potentially persistent peripheral nerve injury associated with these agents [36,86]. Importantly, peripheral neuropathy accounted for the largest share of neurological reports overall, highlighting its relevance as the most clinically impactful neurological manifestation of FQN toxicity and supporting its prioritisation in post-marketing safety surveillance and prescribing considerations.

*Discussions concerning disproportionality analysis*

The disproportionality analysis reveals clear drug-specific neurological risk profiles for Ciprofloxacin, Levofloxacin, and Moxifloxacin, in concordance with previously published literature [32,43,46,86]. Peripheral neuropathy emerges as the strongest and most consistent neurological association for Levofloxacin and Moxifloxacin, and this is supported by their markedly elevated IC values (Levofloxacin IC 4.32; Moxifloxacin IC 4.20) and very high RORs (Levofloxacin ROR 21.12; Moxifloxacin ROR 19.55). Ciprofloxacin shows a slightly lower (but still substantial) association (IC 3.59; ROR 12.67), confirming its relevance as a contributor to peripheral nerve toxicity [36,86,87].

- Ciprofloxacin

**Figure 10:**
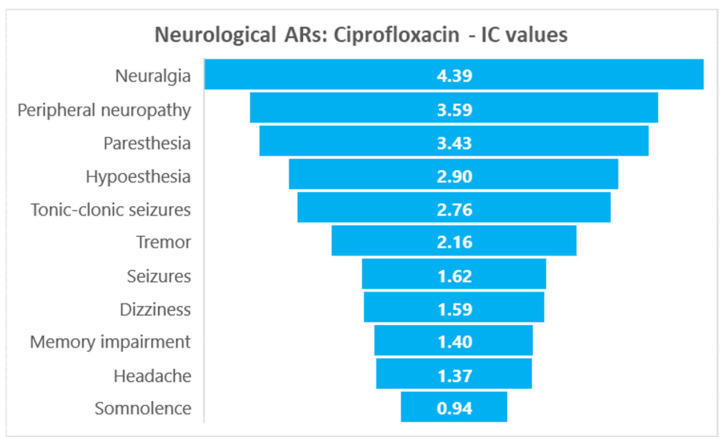
Information Component (IC) values for neurological ARs associated with Ciprofloxacin. The bar chart presents IC estimates for individual neurological ARs, ranked by signal strength in descending order. Positive IC values indicate a higher-than-expected reporting frequency of the drug–event combination compared with the background database.

Ciprofloxacin demonstrates the strongest signals for neuralgia (IC 4.39; ROR 21.90) and paraesthesia (IC 3.43; ROR 11.45) (**Figure 10**, **Table 5**), reflecting a broader sensory-disturbance profile than that observed for Levofloxacin or Moxifloxacin.

The elevated disproportionality measures suggest a pattern of widespread peripheral sensory involvement [46]. Also, Ciprofloxacin shows a broad neurological signal, with particularly elevated disproportionality values for neuralgia (IC 4.39; ROR 21.90), paraesthesia (IC 3.43; ROR 11.45), and peripheral neuropathy (IC 3.59; ROR 12.67). The strong associations highlight a consistent pattern of peripheral sensory disturbances, while additional signals such as hypoesthesia (IC 2.90; ROR 7.75) and tremor (IC 2.16; ROR 4.58) further reinforce its centrally mediated neurotoxicity profile. More common but less specific manifestations, such as dizziness (IC 1.59; ROR 3.11) and headache (IC 1.37; ROR 2.67), remain prominent, indicating frequent but milder CNS involvement. The moderate signal for seizure-related events, including seizures (IC 1.62; ROR 3.12) and tonic–clonic seizures (IC 2.76; ROR 6.86), confirms that convulsive manifestations are reported but do not represent the strongest neurological associations for Ciprofloxacin.

Existing literature supports the sensory-dominant neurological profile observed for Ciprofloxacin in this analysis. Clinical reviews and pharmacovigilance studies consistently report paraesthesia, neuralgia, and peripheral neuropathic symptoms as common neurological ARs associated with Ciprofloxacin, indicating heightened peripheral sensory nerve involvement [46,70]. Epidemiological evidence further corroborates an increased risk of peripheral neuropathy following Ciprofloxacin exposure, supporting the robustness of this signal in clinical practice [36,86]. Our results are biologically plausible given Ciprofloxacin’s documented CNS excitatory effects, which include interference with GABAergic neurotransmission; this may also account for associated tremor, dizziness, and headache [32,43]. Although seizure-related events have been reported, they appear less frequent and typically context-dependent, suggesting that they are secondary rather than dominant neurological manifestations [44,45].

- Levofloxacin

**Figure 11:**
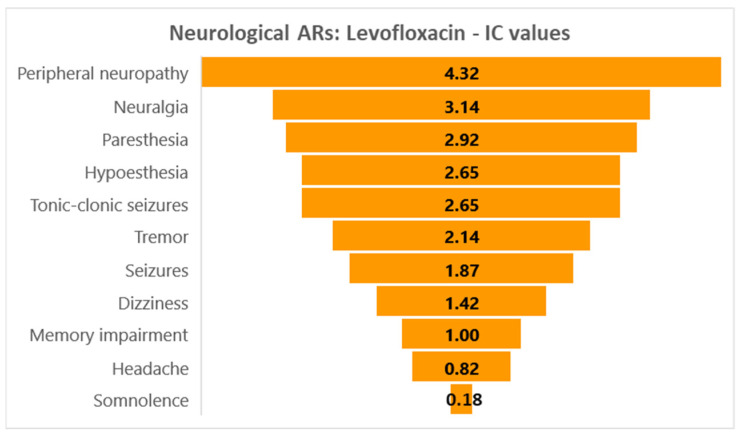
Information Component (IC) values for neurological ARs associated with Levofloxacin. The figure displays IC estimates for reported neurological ARs, ordered by decreasing magnitude. Uniformly positive IC values reflect systematic disproportional reporting, with higher values indicating stronger safety signals.

Levofloxacin exhibits the highest overall neurological risk intensity, as indicated by the clustering of elevated sensory-related IC and ROR values. In addition to peripheral neuropathy, the drug shows strong associations with neuralgia (IC 3.14; ROR 9.01), paraesthesia (IC 2.92; ROR 7.71), and hypoesthesia (IC 2.65; ROR 6.38) (**Figure 11**, **Table 6**).

The consistent pattern across multiple sensory pathways highlights a more diffuse neurological toxicity profile. Additional strong associations include neuralgia (IC 3.14; ROR 9.01), paraesthesia (IC 2.92; ROR 7.71), and hypoesthesia (IC 2.65; ROR 6.38), which form a cluster of elevated sensory-related signals. Central manifestations such as dizziness (IC 1.42; ROR 2.69), tremor (IC 2.14; ROR 4.46), and seizures (IC 1.87; ROR 3.69) also show significant associations, although they are weaker than those for the peripheral findings. The minimal signal for somnolence (IC 0.18; ROR 1.13) suggests that sedation or sleepiness is not a key neurological feature of Levofloxacin.

The available literature provides evidence to support the high neurological risk associated with Levofloxacin, particularly its prominent involvement of peripheral sensory pathways. Reviews and pharmacovigilance studies consistently report peripheral neuropathy, paraesthesia, neuralgia, and hypoesthesia as key neurological adverse reactions associated with Levofloxacin, indicating diffuse sensory nerve toxicity rather than isolated effects [46,70]. Large epidemiological analyses provide further confirmation of a strong association between Levofloxacin exposure and peripheral neuropathy, improving the clinical relevance of the results [36,86]. Central neurological manifestations such as dizziness, tremor, and seizures have also been documented, though they occur less frequently than peripheral sensory disturbances and likely reflect CNS excitatory mechanisms [32,43,45]. In contrast, somnolence appears minimally represented, suggesting that sedative effects are not a defining feature of Levofloxacin-associated neurotoxicity [46].

- Moxifloxacin

**Figure 12:**
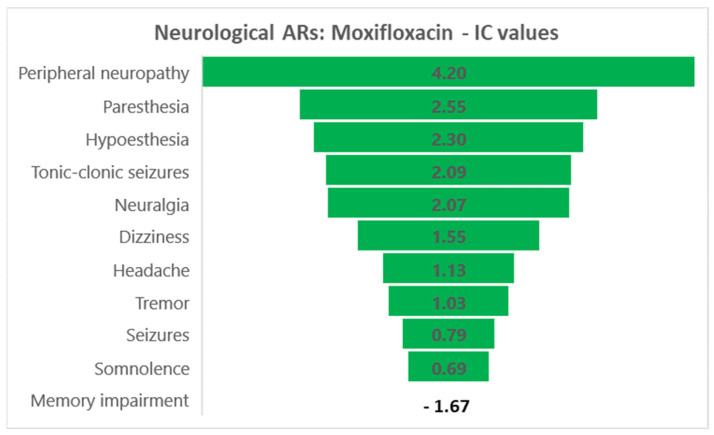
Information Component (IC) values for neurological adverse reactions (ARs) associated with Moxifloxacin. The figure shows IC estimates for neurological ARs, with positive values indicating disproportional reporting relative to the reference database and highlighting compound-specific neurotoxic signal patterns.

Although Moxifloxacin accounts for fewer neurological reports overall, it shows highly concentrated peripheral neurotoxicity signals (IC 4.20; ROR 19.55) (**Figure 12**, **Table 7**), closely paralleling the pattern observed for Levofloxacin.

Strong associations were observed for paraesthesia (IC 2.55; ROR 5.99) and hypoesthesia (IC 2.30; ROR 5.02), highlighting a focused but potent pattern of peripheral nerve involvement. Central symptoms such as dizziness (IC 1.55; ROR 3.01) and headache (IC 1.13; ROR 2.24) remain moderately associated, whereas tremor (IC 1.03; ROR 2.05) shows only a weak signal. Notably, memory impairment displays a negative signal (IC –1.67; ROR 0.31), indicating that there is no disproportionate reporting and suggesting that memory-related complaints are unlikely to be associated with Moxifloxacin exposure. Seizure-related events demonstrate only minimal association, reflected by low disproportionality measures (seizures: IC 0.79; ROR 1.74; tonic–clonic seizures: IC 2.09; ROR 4.28), further indicating that convulsive manifestations represent a comparatively weaker component of the neurological safety profile.

Although Moxifloxacin generated fewer neurological reports overall, available evidence supports a focused yet potent peripheral neurotoxicity profile. Pharmacovigilance reviews and epidemiological data consistently associate Moxifloxacin with peripheral neuropathy, paraesthesia, and hypoesthesia, indicating preferential involvement of peripheral sensory nerves rather than diffuse CNS toxicity [46,70]. Population-based studies confirm that Moxifloxacin, like other later-generation FQNs, carries a clinically relevant risk of peripheral neuropathy, despite having lower overall exposure volumes [36,86]. Central neurological ARs, such as dizziness and headache, have been described but appear less prominent. In contrast, tremor and seizure-related events are reported infrequently, supporting a comparatively weaker central excitatory profile [32,43,45]. The absence of consistent memory-related signals in prior studies aligns with the lack of disproportionate reporting for cognitive impairment observed in this analysis [46].

##### Psychiatric ARs

*Discussions concerning descriptive analysis*

Psychiatric ARs displayed distinct, drug-specific reporting patterns that further add to the non-interchangeability of the evaluated FQNs. Anxiety emerged as the dominant psychiatric manifestation for both Ciprofloxacin and Moxifloxacin. In contrast, Levofloxacin was characterised by a unique predominance of insomnia, a pattern previously reported in pharmacovigilance analyses and clinical case series [38,39,58]. The obtained results suggest differential modulation of central neurochemical pathways among individual FQNs, potentially reflecting variations in GABAergic interference, excitatory neurotransmission, or circadian regulation [32,38]. Although severe psychiatric manifestations such as psychotic disorders and suicidal ideation occurred less frequently, their consistent presence across all three FQNs highlights their clinical relevance, particularly given their potential severity, abrupt onset, and inclusion in regulatory safety warnings [34,55]. The overall distribution of psychiatric ARs indicates that affective and sleep-related disturbances represent the core psychiatric expression of FQN-associated neurotoxicity. At the same time, more psychotic phenomena appear as less common but clinically significant events [38,56]. Collectively, the descriptive analysis highlights the importance of active psychiatric symptom monitoring during FQN therapy, especially in susceptible populations or in patients with pre-existing mental health conditions [34,58].

*Discussions concerning disproportionality analysis*

Psychiatric manifestations displayed notable variability across the three FQNs. Anxiety emerged as the most frequently reported psychiatric AR for both Ciprofloxacin (**Figure 6a**) and Moxifloxacin (**Figure 6c**), whereas insomnia predominated for Levofloxacin (**Figure 6b**). The patterns indicate that, while all three FQNs influence central neurotransmitter pathways, each drug may exert a distinct modulatory impact on affective or cognitive domains.

- Ciprofloxacin

**Figure 13:**
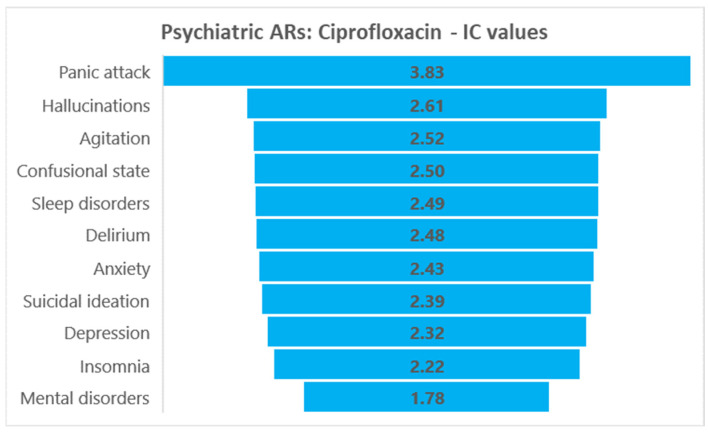
Information Component (IC) values for psychiatric adverse reactions (ARs) associated with Ciprofloxacin. The bar chart displays IC estimates for each reported psychiatric AR, ranked by signal strength in descending order. Positive IC values indicate a higher-than-expected reporting frequency of the drug–event combination compared with the background database.

**Figure 13** illustrates the IC values for psychiatric ARs reported in association with Ciprofloxacin, highlighting a consistent pattern of positive disproportionality across all evaluated results.

All IC estimates are >0, indicating higher-than-expected co-reporting of Ciprofloxacin with these psychiatric events compared with the background reporting in the database. Panic attacks show the strongest disproportionality signal (IC 3.83; ROR 14.66), indicating a robust, non-random association between Ciprofloxacin exposure and this AR (**Table 5**). Hallucinations (IC 2.61; ROR 6.24), agitation (IC 2.52; ROR 5.83), confusional state (IC 2.50; ROR 5.81), sleep disorders (IC 2.49; ROR 5.70), and delirium (IC 2.48; ROR 5.64) also demonstrate substantial disproportionality. The clustering of these ARs suggests a broader neuropsychiatric signal profile, encompassing perceptual disturbances, alterations in consciousness, and psychomotor agitation. The magnitude and consistency of IC and ROR values across these outcomes indicate a stable signal that is unlikely to be explained solely by reporting bias.

Anxiety, suicidal ideation, depression, and insomnia exhibit moderately elevated disproportionality signals, with consistently positive IC values and RORs well above unity, supporting clinically relevant associations with Ciprofloxacin. Previously published case reports support our results [40,88,89,90]. The identification of suicidal ideation among these outcomes is particularly noteworthy given its clinical severity. In contrast, psychotic disorders show the lowest IC and ROR estimates, suggesting a weaker signal that may be attenuated by diagnostic heterogeneity or classification breadth.

- Levofloxacin

**Figure 14:**
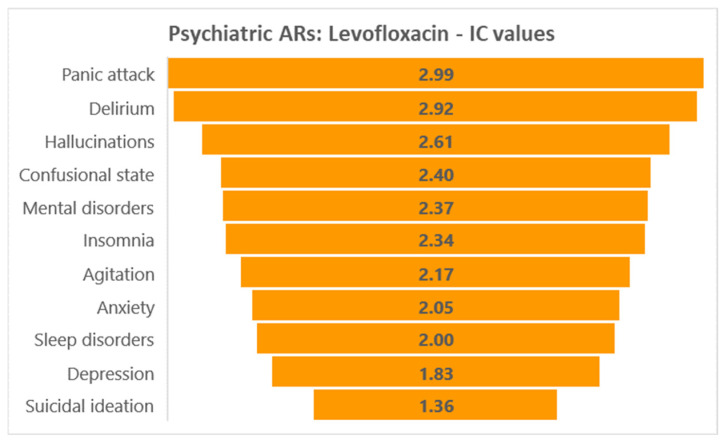
Information Component (IC) values for psychiatric adverse reactions (ARs) associated with Levofloxacin. Shown are IC estimates for individual psychiatric ARs, ordered by decreasing magnitude. Uniformly positive IC values suggest consistent disproportional reporting.

As shown in **Figure 14**, all evaluated psychiatric ARs associated with Levofloxacin present positive IC values, reflecting a systematic pattern of disproportional reporting.

The strongest signals are observed for panic attacks (IC = 2.99; ROR = 8.08) and delirium (IC = 2.92; ROR = 7.71) (**Table 6**), reflecting robust associations supported by concordant elevations in both disproportionality metrics. Hallucinations, confusional state, mental disorders, and insomnia also exhibit substantial IC estimates (IC > 2.3) accompanied by RORs well above unity, reinforcing the presence of a stable neuropsychiatric safety signal. Moderately elevated IC and ROR values are noted for agitation, anxiety, sleep disorders, and depression, suggesting clinically relevant but less pronounced associations. In contrast, suicidal ideation shows the lowest disproportionality (IC = 1.36; ROR = 2.58), indicating a weaker—yet still positive—signal. Clinical cases in the literature support the observed psychiatric disproportionality signals for Levofloxacin, with reports by Pires A. et al. (2011) [91] describing rapid-onset agitation, delirium, and insomnia, while Odeh M. et al. (2019) [92] identify Levofloxacin as an under-recognised contributor to drug-induced delirium, particularly among elderly patients. The case report of Kandasamy A. and Srinath D. (2012) [39] supports our results of insomnia dominance with Levofloxacin.

Overall, the concordance between IC and ROR values across outcomes supports the robustness of the observed psychiatric safety profile for Levofloxacin.

- Moxifloxacin

**Figure 15:**
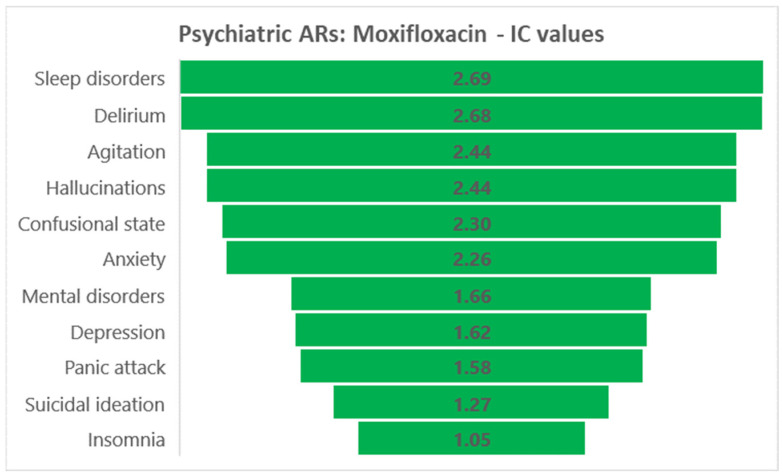
Information Component (IC) values for psychiatric adverse reactions (ARs) associated with Moxifloxacin. The figure presents IC estimates for reported psychiatric ARs, with positive values indicating disproportional reporting relative to the reference database.

**Figure 15** shows uniformly positive IC values for psychiatric ARs associated with Moxifloxacin, indicating consistent disproportional reporting.

The strongest signals are observed for sleep disorders (IC = 2.69; ROR = 6.52) and delirium (IC = 2.68; ROR = 6.44), reflecting robust associations supported by concordant elevations in both disproportionality measures (**Table 7**). In the clinical field, Güler S. and Kocaşaban D. (2023) [64] published a case report of an elderly patient with Moxifloxacin-induced mental status changes (acute confusion and delirium). Previously, another FAERS study reported that Moxifloxacin was most strongly linked to delirium among FQNs [55]. Agitation and hallucinations (both IC = 2.44; ROR ≈ 5.47–5.49), as well as confusional state and anxiety (IC > 2.25), further reinforce a consistent neuropsychiatric safety signal. For example, visual hallucinations associated with Moxifloxacin were reported by Uz B. (2020) [35]. Lower—but still positive—IC and ROR values are noted for mental disorders, depression, and panic attacks, suggesting weaker (yet persistent) associations. Suicidal ideation (IC = 1.27; ROR = 2.42) and insomnia (IC = 1.05; ROR = 2.09) display the lowest disproportionality estimates, indicating comparatively modest signals. Overall, the concordance between IC and ROR across outcomes supports a stable psychiatric safety profile for Moxifloxacin, although with generally lower signal strength than observed for Ciprofloxacin or Levofloxacin.

Across the three FQNs, psychiatric ARs demonstrated consistently positive disproportionality signals, with both IC and ROR values supporting non-random associations, in concordance with other previous data [38,55]. Ciprofloxacin showed the strongest overall signals, particularly for panic attacks, hallucinations, agitation, and confusional states, reflected by high IC estimates and markedly elevated RORs, indicating a robust psychiatric safety signal. Levofloxacin displayed a broadly similar profile, with pronounced disproportionality for panic attacks and delirium and moderate-to-high signals across most other psychiatric outcomes, suggesting a stable but generally less intense pattern than Ciprofloxacin. Moxifloxacin was associated with uniformly positive but overall lower IC and ROR values, with the strongest signals observed for sleep disorders and delirium, indicating a comparatively weaker (yet consistent) psychiatric signal. The concordance between IC and ROR values across all three FQNs highlights a class-related neuropsychiatric risk, with variation in signal strength among individuals. Also, all signals discussed exhibit IC025 > 0, indicating statistical stability.

The obtained results support a class-related neuropsychiatric risk among FQNs, with Ciprofloxacin exhibiting the strongest signals, Levofloxacin exhibiting an intermediate profile, and Moxifloxacin exhibiting comparatively weaker (but consistent) associations.

#### 3.5.3. Seriousness Classification for All AR Reports Submitted to the FAERS Database

Over the examined period, the number of serious reports has remained consistently high, accounting for around half of all submitted ARs each year (**Figure 7**). Such a pattern indicates that FAERS tends to capture events with substantial clinical impact (e.g., hospitalisation, life-threatening episodes, disability, or death), as less severe cases are often under-reported. The upward trend over several years suggests increasing awareness, improved reporting efficiency, or genuine expansion of severe safety outcomes in clinical use.

Also, a large FAERS characterisation study analysing over 2 million serious reports (2016–2023) shows that FAERS contains a very high volume of serious outcomes, including hospitalisation, life-threatening events, disability, and death [93]. However, FAERS data are limited by under-reporting and variability in report completeness, especially for non-serious events [94]. The FDA itself states that FAERS contains ARs received from manufacturers, as required by regulations, especially for serious events, which contributes to the over-representation of serious ARs in FAERS [47].

### 3.6. FDA and EMA Safety Warnings on FQN-Associated Neuropsychiatric Effects

FQNs have been the subject of multiple major safety communications issued by both the FDA and EMA, reflecting growing concern regarding their neuropsychiatric toxicity profile. Over the past decade, regulatory agencies have progressively strengthened warnings based on accumulating post-marketing evidence [6].

In 2013, the FDA required label changes to warn of the risk for possibly permanent nerve damage from FQNs taken by mouth or by injection [87]. Also, in 2016, the FDA released a boxed warning update emphasising disabling and potentially permanent adverse effects involving the CNS, including confusion, agitation, hallucinations, and other psychiatric disturbances. Subsequent updates in 2018 highlighted additional risks such as mental health side effects and severe blood glucose disturbances [6,32]. Similarly, the EMA implemented strengthened safety measures, issuing restrictions on systemic and inhaled FQNs due to long-lasting, potentially irreversible ARs, including psychiatric symptoms, peripheral neuropathy, and musculoskeletal toxicity. The agency also mandated updates to product information and educational materials to ensure that prescribers and patients are informed of these risks [95,96].

The FDA and EMA warnings indicate that neuropsychiatric ARs are well-documented in clinical and pharmacovigilance data and are recognised at the highest regulatory level, supporting the clinical importance and public health relevance of the safety signals identified in our study.

### 3.7. Clinical Relevance and Implications

Our results have substantial clinical relevance, particularly given the widespread prescribing of FQNs for common community and hospital infections. The consistent detection of neuropsychiatric ARs across Ciprofloxacin, Levofloxacin, and Moxifloxacin improves the clinical importance of these events; it supports the existing literature identifying central and peripheral neurotoxicity as a recognised safety concern associated with the FQNs class. Prior reviews have emphasised that FQNs may induce excitatory CNS effects through GABA receptor antagonism and NMDA receptor overstimulation, which biologically supports many of the manifestations observed in this study, including anxiety, insomnia, hallucinations, and seizures [32,68,69,70].

From a prescribing perspective, our results highlight the need for individualised risk–benefit assessment. The strong disproportionality signals observed for peripheral neuropathy, particularly with Levofloxacin and Moxifloxacin, align with recent epidemiological and pharmacovigilance evaluations showing increased peripheral nerve toxicity associated with these FQNs [36,87]. It highlights the need for caution in patients with predisposing factors such as diabetes, renal impairment, or concurrent neurotoxic medications. Similarly, the prominent association of Ciprofloxacin with centrally mediated symptoms (hallucinations, neuralgia, and paraesthesia) supports earlier evidence identifying Ciprofloxacin and Ofloxacin as the FQNs with the greatest CNS toxicity potential [32,60].

Our study also emphasises the importance of early detection and timely intervention. Case reports consistently describe the onset of acute psychiatric or neurological symptoms within hours to a few days of exposure, with rapid improvement after drug discontinuation [35,39,40,41,61,62,63,64,72]. Clinician awareness is essential to avoid delayed recognition, since many of these ARs (e.g., agitation, confusion, anxiety, derealisation) are misinterpreted as primary psychiatric conditions. Proactive patient counselling can further facilitate early reporting, particularly in older adults, who represent a higher-risk population and account for a substantial proportion of neuropsychiatric reports in this study.

The relevance of neuropsychiatric safety concerns is further highlighted by multiple FDA and EMA warnings addressing the risks associated with systemic FQNs use. Both agencies have issued increasingly stringent safety warnings regarding CNS, psychiatric, and peripheral neuropathic ARs associated with systemic FQN use [8,9,10]. The present study provides updated pharmacovigilance evidence supporting these regulatory actions, accentuating the need to reserve systemic FQNs for cases where no safer alternatives are available, as recommended by both agencies.

From a public health and pharmacovigilance standpoint, this study highlights the importance of continued surveillance and signal detection. The divergence in neuropsychiatric profiles across the three evaluated FQNs suggests that these agents should not be treated as interchangeable from a safety perspective. Rather, drug-specific neuropsychiatric risk signatures (identified here and supported by several large-scale FAERS analyses [56,71]) should guide antibiotic stewardship initiatives and clinical decision-making.

The obtained results carry important implications for future research. The differential patterns observed across Ciprofloxacin, Levofloxacin, and Moxifloxacin point toward distinct mechanisms and pathways that require further elucidation. Recent network-based and molecular studies highlight possible involvement of inflammatory signalling, mitochondrial dysfunction, and protein targets such as HSP90AA1 in FQN-induced neurotoxicity [65,87]. Additional clinical, pharmacogenomic, and mechanistic investigations are needed to identify high-risk populations, clarify causative pathways, and inform personalised prescribing. Improved understanding of the underlying mechanisms, combined with heightened clinical vigilance, may reduce the concern of FQN-related neuropsychiatric toxicity in routine practice.

## 4. Materials and Methods

### 4.1. Study Design

This research was conducted as a retrospective pharmacovigilance study.

### 4.2. Data Source

Data for this study were obtained from the FAERS, a spontaneous reporting database used for post-marketing safety surveillance of drugs and therapeutic biologics. FAERS follows the ICH E2B(R3) standard for pharmacovigilance reporting and uses the Medical Dictionary for Regulatory Activities (MedDRA) for coding all adverse events. Data were extracted from the FAERS Public Dashboard in January 2026. As the Dashboard reflects a dynamic, continuously updated database, all reported counts correspond to values retrieved during this extraction period. Minor discrepancies between descriptive statistics and disproportionality parameters reflect sequential extraction sessions conducted within this timeframe and are not expected to affect signal estimates materially.

The dataset includes mandatory reports from manufacturers and voluntary reports from healthcare professionals and consumers.

The analysis covered 16 years (2010–2025) and included all reports in which Ciprofloxacin, Levofloxacin, or Moxifloxacin were listed as the primary suspect drug. Only reports explicitly coded with MedDRA terms corresponding to neurological or psychiatric manifestations were retained for analysis.

### 4.3. Selection of Neuropsychiatric ARs

Neuropsychiatric adverse reactions (ARs) were identified using MedDRA terminology and the Standardised MedDRA Query (SMQ) for Peripheral Neuropathy. Terms were grouped into two categories:

- Neurological ARs: dizziness, headache, hypoesthesia, memory impairment, neuralgia, paraesthesia, peripheral neuropathy, seizures, somnolence, tonic–clonic seizures, and tremor.
- Psychiatric ARs: agitation, anxiety, confusional state, delirium, depression, hallucinations, insomnia, panic attack, psychotic disorders, sleep disorders, and suicidal ideation.

Duplicate reports were identified using patient age, sex, event date, and case identifiers, and removed in accordance with the FDA deduplication guidelines.

### 4.4. Descriptive Statistical Analysis

Descriptive statistics were used to summarise annual reporting trends (2010–2025), demographic profiles (age and sex), and the frequency and distribution of neurological and psychiatric ARs for each FQN. Frequencies and percentages were calculated relative to the total number of neuropsychiatric AR reports for each specific drug. Graphical representations were generated to illustrate reporting dynamics and comparative distributions among the selected FQNs. Data management, tabulation, and graphical analyses were performed using Microsoft Excel (Microsoft Corporation, Redmond, WA, USA).

### 4.5. Disproportionality Analysis

To identify potential safety signals, three complementary disproportionality metrics were applied [49,50,51,52]:

1. Information Component (IC)
  - It is a Bayesian measure developed by the WHO-UMC;
  - IC025, the lower limit of the 95% credibility interval, was used to determine significance;
  - IC025 > 0 indicates a statistically robust signal.

The IC and its lower 95% credibility limit (IC025) were calculated in Microsoft Excel using the Bayesian Confidence Propagation Neural Network (BCPNN) formulas described by the World Health Organisation-Uppsala Monitoring Centre (WHO-UMC).

2. Proportional Reporting Ratio (PRR)
  - Calculates the relative frequency of an AR for a specific drug compared with all other drugs in the database;
  - PRR025, the lower 95% confidence limit, was used for significance assessment;
  - PRR025 > 1 was considered indicative of a signal.
3. Reporting Odds Ratio (ROR)
  - Assesses the odds of reporting an AR with a given drug compared to all others;
  - ROR > 1 suggests disproportionate reporting, with higher values reflecting stronger associations.

PRR and ROR disproportionality analyses were conducted using MedCalc statistical software (MedCalc Software Ltd., Ostend, Belgium; version 23.2.6) [53].

For all disproportionality calculations, the following totals were used [97]:

- Ntot: Complete FAERS database reports for the 2010–2025 period;
- Ndrug: Total number of reports per FQN;
- Nadr: Number of reports per AR term;
- Ncomb: Drug–event pair count.

Signal detection was performed according to internationally accepted methodologies used by the European Medicines Agency (EMA), World Health Organisation-Uppsala Monitoring Centre (WHO-UMC), and the FDA in pharmacovigilance practice [50,54,98].

## 5. Strengths and Limitations of the Study

### 5.1. Strengths

The study is strengthened by its use of a large, long-term pharmacovigilance dataset, which provides sufficient statistical power and temporal scope to identify rare yet clinically relevant neuropsychiatric ARs. The combined application of multiple disproportionality measures (IC, PRR, and ROR) enhances the robustness of signal detection. At the same time, the standardised, MedDRA-based selection of ARs improves reproducibility and comparability with existing research. Moreover, the concurrent assessment of three widely prescribed FQNs within a unified analytical framework enables clear differentiation between class-wide effects and compound-specific risk profiles. Integrating pharmacovigilance data with clinical case reports and regulatory safety communications adds to biological plausibility and clinical relevance, thereby supporting the applicability of the results to prescribing and risk assessment.

### 5.2. Limitations

However, the present study has several inherent limitations related to the use of FAERS data. Since FAERS is a spontaneous reporting system, ARs are subject to substantial under-reporting, variable data quality, and possible duplication, which limits the reliability of the dataset. IC025 values were calculated using custom spreadsheet formulas rather than validated pharmacovigilance software, which may limit their direct comparability with WHO-UMC signal detection outputs. The absence of exposure denominators and clinical details, such as dosage, treatment duration, comorbidities, or concomitant medications, prevents the calculation of true incidence rates and limits the control of confounding factors. Additionally, MedDRA coding depends on reporter interpretation, which may lead to inconsistent classification of neuropsychiatric events. Because disproportionality analyses and retrospective spontaneous-report data cannot establish causality, the results should be interpreted as signals rather than confirmatory.

## 6. Conclusions

The present pharmacovigilance analysis provides a comprehensive, comparative characterisation of the neuropsychiatric safety profiles of three widely prescribed systemic FQNs (Ciprofloxacin, Levofloxacin, and Moxifloxacin) using FAERS data spanning a period of 16 years. The results demonstrate that neuropsychiatric ARs represent a clinically relevant and persistent component of FQN toxicity, with consistent reporting across neurological and psychiatric domains. Importantly, the results show that these ARs are not uniformly distributed across the FQN class but exhibit distinct, compound-specific risk profiles, evidencing the non-interchangeability of individual agents from a safety perspective.

Peripheral neuropathy appeared as the most robust and consistent neurological safety signal, particularly for Levofloxacin and Moxifloxacin; this was supported by markedly elevated IC and ROR values and stable lower confidence bounds. In contrast, Ciprofloxacin demonstrated stronger associations with centrally mediated neurological manifestations, including neuralgia, paraesthesia, and headache. Psychiatric ARs also displayed drug-specific patterns, with anxiety predominating for Ciprofloxacin and Moxifloxacin, while insomnia represented the dominant psychiatric signal for Levofloxacin. Across all three FQNs, disproportionality analyses revealed statistically stable signals for several severe neuropsychiatric outcomes, including delirium, hallucinations, panic attacks, and suicidal ideation, adding to their clinical and regulatory relevance.

Generally, the obtained data strengthen existing regulatory warnings and clinical observations by providing quantitative, comparative evidence from post-marketing surveillance. The results confirm that FQNs are not interchangeable with respect to neuropsychiatric safety. Clinically, the findings highlight the need for individualised risk–benefit assessment when prescribing FQNs, particularly for vulnerable patients. Increased awareness, early detection of neuropsychiatric symptoms, and timely discontinuation may reduce patient harm.

From a pharmacovigilance and public health perspective, the present study highlights the value of large-scale disproportionality analyses in clarifying drug-specific safety signatures that are not always apparent in clinical trials. While causality cannot be established from spontaneous reporting data, the consistency and magnitude of the detected signals support the need for continued surveillance, targeted pharmacoepidemiological studies, and research into mechanisms that will further elucidate the biological pathways underlying FQN-associated neuropsychiatric toxicity. Ultimately, integrating drug-specific neuropsychiatric risk profiles into antimicrobial stewardship strategies may lead to safer, more rational use of FQNs in clinical practice.

## Figures and Tables

**Table 1:** General characteristics of recently approved and regionally authorised fluoroquinolones (FQNs) for systemic use; the table summarises FQNs that have received regulatory approval for systemic administration by the U.S. Food and Drug Administration (FDA), the European Medicines Agency (EMA), or national regulatory authorities, as well as selected newer agents approved regionally outside the EU or USA; (Ref. = reference).

Fluoroquinolone	Year of Approval	Indication	Route of Administration	Antibacterial Spectrum	Observations	Ref.
Delafloxacin	2017 2019	Community-acquired pneumonia Acute skin and skin structure infections	Oral Parenteral	Broad spectrum, Gram-positive bacteria, including methicillin-resistant *Staphylococcus aureus* (MRSA), and Gram-negative bacteria	FDA- and EMA-approved	[13,14,15]
Lascufloxacin	2019 2022	Respiratory system infections (ear, nose, and throat infections, pneumonia, etc.)	Oral	*Haemophilus influenzae*, *Legionella pneumophila*, *Moraxella catarrhalis*, *Mycoplasma pneumoniae*, *Prevotella*, *Streptococcus pneumoniae*	Approved in Japan Approved in China	[16,17,18]
Levonadifloxacin	2020	Diabetic foot infections Acute skin and skin structure infections with concomitant bacteraemia	Oral Parenteral	Broad spectrum, including MRSA and *Staphylococcus aureus*, resistant to other FQNs Atypical bacteria Anaerobic bacteria	Approved in India	[19,20]
Nemonoxacin	2014 2016	Community-acquired pneumonia	Oral Parenteral	Typical and atypical respiratory pathogens, especially resistant Gram-positive cocci (*MRSA* and *Streptococcus pneumoniae* resistant to penicillins), *Clostridium difficile, Helicobacter pylori* Vancomycin-*resistant pathogens*	Approved in Taiwan Approved in China	[21,22,23,24,25,26]
Sitafloxacin	2008 2012	Urinary tract infections Respiratory infections	Oral	Broad spectrum: Gram-positive and Gram-negative bacteria, anaerobic bacteria, and atypical pathogens resistant to other FQNs	Approved in Japan Approved in Thailand	[27,28]
Zabofloxacin	2015	Acute bacterial exacerbations of chronic obstructive pulmonary disease	Oral	Broad spectrum, especially against respiratory pathogens (*Haemophilus influenzae*, *Moraxella carrhalis* or *Staphylococcus aureus)* *Neisseria gonorrhoeae*	Approved in South Korea, the Middle East and North African countries	[29,30,31]

**Table 2:** Distribution of neuropsychiatric AR reports by age group for Ciprofloxacin, Levofloxacin, and Moxifloxacin in the FAERS database (2010–2025). Values are presented as percentages within each FQN, with corresponding report counts, including cases with unknown age.

FQNs	0–1 Months	%	2 Months–2 Years	%	3–11 Years	%	12–17 Years	%	18–64 Years	%	65–85 Years	%	>85 years	%	Unknown	%
Ciprofloxacin	41	47.67	179	60.27	412	55.23	477	50.16	18,121	44.77	10,944	42.14	1610	42.79	7269	30.70
Levofloxacin	42	48.84	98	33.00	247	33.11	376	39.54	18,346	45.33	12,956	49.88	1936	51.45	14,423	60.92
Moxifloxacin	3	3.49	20	6.73	87	11.66	98	10.30	4009	9.90	2072	7.98	217	5.77	1985	8.38
**Total**	**86**		**297**		**746**		**951**		**40** **,** **476**		**25,972**		**3763**		**23,677**	

**Table 3:** Neurological adverse reactions (ARs) reported in association with Ciprofloxacin, Levofloxacin, and Moxifloxacin in the FAERS database (2010–2025). Absolute numbers of reported events are shown, with the highest values highlighted in bold.

Neurological ARs	Ciprofloxacin	%	Levofloxacin	%	Moxifloxacin	%	Total	%
Dizziness	1794	15.21	1975	15.15	378	19.30	4147	15.48
Headache	**1991**	16.88	1685	12.92	366	18.68	4042	15.09
Hypoesthesia	1365	11.57	1424	10.92	196	10.01	2985	11.14
Memory impairment	462	3.92	436	3.34	12	0.61	910	3.40
Neuralgia	644	5.46	336	2.58	28	1.43	1008	3.76
Paraesthesia	1969	16.69	1714	13.15	232	11.84	3915	14.61
Peripheral neuropathy	1435	12.16	**2945**	22.59	**476**	24.30	**4856**	18.12
Seizures	607	5.15	893	6.85	74	3.78	1574	5.87
Somnolence	462	3.92	337	2.58	84	4.29	883	3.30
Tonic–clonic seizures	190	1.61	219	1.68	26	1.33	435	1.62
Tremor	878	7.44	1073	8.23	87	4.44	2038	7.61
**Total**	**11,797**	**100**	**13,037**	**100**	**1959**	**100**	**26,793**	**100**

**Table 4:** Psychiatric adverse reactions (ARs) reported in association with Ciprofloxacin, Levofloxacin, and Moxifloxacin in the FAERS database (2010–2025). Absolute numbers of reported events are shown, with the highest values highlighted in bold.

Psychiatric ARs	Ciprofloxacin	%	Levofloxacin	%	Moxifloxacin	%	Total	%
Agitation	395	4.46	384	4.18	81	5.92	860	4.43
Anxiety	**1971**	22.25	1798	19.59	**364**	26.61	**4133**	21.30
Confusional state	1059	11.96	1232	13.42	201	14.69	2492	12.84
Delirium	225	2.54	378	4.12	56	4.09	659	3.40
Depression	1346	15.20	1183	12.89	179	13.08	2708	13.96
Hallucinations	535	6.04	660	7.19	103	7.53	1298	6.69
Insomnia	1501	16.95	**2016**	21.96	145	10.60	3662	18.87
Panic attack	612	6.91	424	4.62	28	2.05	1064	5.48
Psychotic disorders	186	2.10	346	3.77	37	2.70	569	2.93
Sleep disorder	478	5.40	424	4.62	119	8.70	1021	5.26
Suicidal ideation	549	6.20	334	3.64	55	4.02	938	4.83
**Total**	**8857**	**100**	**9179**	**100**	**1368**	**100**	**19,404**	**100**

**Table 5:** Ciprofloxacin—neuropsychiatric adverse drug reactions (ARs) positive association (IC = Information Component, PRR = Proportional Reporting Ratio, ROR = Reporting Odds Ratio).

No.	Ars	IC	IC025	PRR	PRR025	ROR
1	Agitation	2.52	2.37	5.78	5.23	5.83
2	Anxiety	2.43	2.36	5.44	5.20	5.66
3	Confusional state	2.50	2.41	5.68	5.35	5.81
4	Delirium	2.48	2.29	5.62	4.93	5.64
5	Depression	2.32	2.24	5.04	4.77	5.18
6	Dizziness	1.59	1.52	3.02	2.88	3.11
7	Hallucinations	2.61	2.49	6.16	5.66	6.24
8	Headache	1.37	1.30	2.59	2.48	2.67
9	Hypoesthesia	2.90	2.82	7.52	7.13	7.75
10	Insomnia	2.22	2.15	4.69	4.46	4.83
11	Memory impairment	1.40	1.26	2.64	2.41	2.66
12	Mental disorders	1.78	1.57	3.45	2.99	3.46
13	Neuralgia	4.39	4.27	21.55	19.94	21.90
14	Panic attack	3.83	3.71	14.45	13.35	14.66
15	Paraesthesia	3.43	3.36	10.92	10.45	11.45
16	Peripheral neuropathy	3.59	3.52	12.24	11.62	12.67
17	Seizures	1.62	1.50	3.09	2.85	3.12
18	Sleep disorders	2.49	2.35	5.65	5.16	5.70
19	Somnolence	0.94	0.81	1.92	1.76	1.93
20	Suicidal ideation	2.39	2.26	5.26	4.84	5.32
21	Tonic–clonic seizures	2.76	2.55	6.83	5.92	6.86
22	Tremor	2.16	2.00	4.50	4.21	4.58

**Table 6:** Levofloxacin—neuropsychiatric adverse drug reactions (ARs) positive association (IC = Information Component, PRR = Proportional Reporting Ratio, ROR = Reporting Odds Ratio).

No.	Ars	IC	IC025	PRR	PRR025	ROR
1	Agitation	2.17	2.00	4.53	4.10	4.55
2	Anxiety	2.05	1.97	4.16	3.97	4.18
3	Confusional state	2.40	2.30	5.31	5.02	5.35
4	Delirium	2.92	2.75	7.65	6.91	7.71
5	Depression	1.83	1.73	3.56	3.37	3.58
6	Dizziness	1.42	1.34	2.68	2.56	2.69
7	Hallucinations	2.61	2.48	6.14	5.69	6.19
8	Headache	0.82	0.74	1.77	1.68	1.77
9	Hypoesthesia	2.65	2.56	6.32	6.01	6.38
10	Insomnia	2.34	2.26	5.08	4.87	5.12
11	Memory impairment	1.00	0.84	2.01	1.83	2.01
12	Mental disorders	2.37	2.19	5.20	4.68	5.22
13	Neuralgia	3.14	2.96	8.93	8.02	9.01
14	Panic attack	2.99	2.83	8.02	7.29	8.08
15	Paraesthesia	2.92	2.84	7.65	7.30	7.71
16	Peripheral neuropathy	4.32	4.26	20.62	19.90	21.12
17	Seizures	1.87	1.76	3.67	3.44	3.69
18	Sleep disorders	2.00	1.84	4.03	3.67	4.05
19	Somnolence	0.18	0.00	1.13	1.02	1.13
20	Suicidal ideation	1.36	1.18	2.57	2.31	2.58
21	Tonic–clonic seizures	2.65	2.43	6.35	5.56	6.39
22	Tremor	2.14	2.04	4.44	4.18	4.46

**Table 7:** Moxifloxacin—neuropsychiatric adverse drug reactions (ARs) positive association (IC = Information Component, PRR = Proportional Reporting Ratio, ROR = Reporting Odds Ratio).

No.	ARs	IC	IC025	PRR	PRR025	ROR
1	Agitation	2.44	2.07	5.43	4.37	5.47
2	Anxiety	2.26	2.09	4.79	4.33	4.96
3	Confusional state	2.30	2.06	4.92	4.29	5.01
4	Delirium	2.68	2.24	6.41	4.93	6.44
5	Depression	1.62	1.37	3.07	2.66	3.11
6	Dizziness	1.55	1.38	2.92	2.65	3.01
7	Hallucinations	2.44	2.12	5.43	4.48	5.49
8	Headache	1.13	0.96	2.19	1.98	2.24
9	Hypoesthesia	2.30	2.07	4.93	4.29	5.02
10	Insomnia	1.05	0.78	2.08	1.77	2.09
11	Memory impairment	−1.67	−2.60	0.32	0.18	0.31
12	Mental disorders	1.66	1.12	3.15	2.29	3.16
13	Neuralgia	2.07	1.45	4.20	2.90	4.21
14	Panic attack	1.58	0.96	2.99	2.07	3.00
15	Paraesthesia	2.55	2.33	5.85	5.16	5.99
16	Peripheral neuropathy	4.20	4.05	18.51	16.96	19.55
17	Seizures	0.79	0.41	1.73	1.38	1.74
18	Sleep disorders	2.69	2.38	6.44	5.39	6.52
19	Somnolence	0.69	0.33	1.61	1.30	1.62
20	Suicidal ideation	1.27	0.83	2.41	1.85	2.42
21	Tonic–clonic seizures	2.09	1.45	4.27	2.91	4.28
22	Tremor	1.03	0.68	2.04	1.66	2.05

## Data Availability

The original contributions presented in this study are included in the article. Further inquiries can be directed to the corresponding author.

## Abbreviations

The following abbreviations are used in this manuscript:

AR(s)	Adverse Reaction(s).
CI	Confidence Interval.
CNS	Central Nervous System.
EMA	European Medicines Agency.
FAERS	Food and Drug Administration Adverse Event Reporting System.
FDA	Food and Drug Administration.
FQN(s)	Fluoroquinolone(s).
GABA	Gamma-Aminobutyric Acid.
IC	Information Component.
IC025	Lower bound of the 95% credibility interval of the Information Component.
MAPK	Mitogen-Activated Protein Kinase.
MDR	Multidrug-Resistant.
MedDRA	Medical Dictionary for Regulatory Activities.
MRSA	Methicillin-resistant *Staphylococcus aureus*.
NMDA	*N*-Methyl-*D*-Aspartate.
NOD	Nucleotide-Binding Oligomerisation Domain.
PN	Peripheral Neuropathy.
PRR	Proportional Reporting Ratio.
PRR025	Lower bound of the 95% confidence interval of the Proportional Reporting Ratio.
PNS	Peripheral Nervous System.
ROR	Reporting Odds Ratio.
SMQ	Standardised MedDRA Query.
UTIs	Urinary Tract Infections.
WHO-UMC	World Health Organisation-Uppsala Monitoring Centre.
